# Linker-free incorporation of carbohydrates into *in vitro* displayed macrocyclic peptides†
†Electronic supplementary information (ESI) available: Primer sequences, additional MALDI-TOF-MS spectra, TIC traces for LC-ESI-MS, and supplementary results on compatibility as noted in the text. See DOI: 10.1039/c6sc04381j


**DOI:** 10.1039/c6sc04381j

**Published:** 2016-10-21

**Authors:** S. A. K. Jongkees, S. Umemoto, H. Suga

**Affiliations:** a Department of Chemistry , Graduate School of Science , The University of Tokyo , 7-3-1 Hongo , 113-0033 Tokyo , Bunkyo-ku , Japan . Email: hsuga@chem.s.u-tokyo.ac.jp; b JST CREST , The University of Tokyo , 7-3-1 Hongo , 113-0033 Tokyo , Bunkyo-ku , Japan

## Abstract

We report a strategy for efficient post-translational modification of a library of ribosomally-translated peptides by activation and elimination of cysteine to dehydroalanine then conjugate addition of a range of exogenous thiols, with an emphasis on carbohydrates. These reactions are selective for cysteine, and do not interfere with amplification of the nucleic acid component of an mRNA-displayed peptide. Furthermore, these reactions are shown to be compatible with two different macrocyclisation chemistries, and when applied to a peptide containing an *N*-terminal cysteine give a ketone that can be functionalised in an orthogonal manner. This new strategy can overcome a limitation of ribosomal translation, providing a means to incorporate untranslatable groups such as carbohydrates in amino acid side chains, and will allow for the ribosomal generation of glycopeptides, requiring only the introduction of a free thiol in the molecule to be incorporated. In combination with *in vitro* selection techniques, this strategy is envisaged to allow the discovery of biologically-active glycopeptides with a near-natural, but hydrolytically stable, thioglycosidic bond.

## Abstract 2

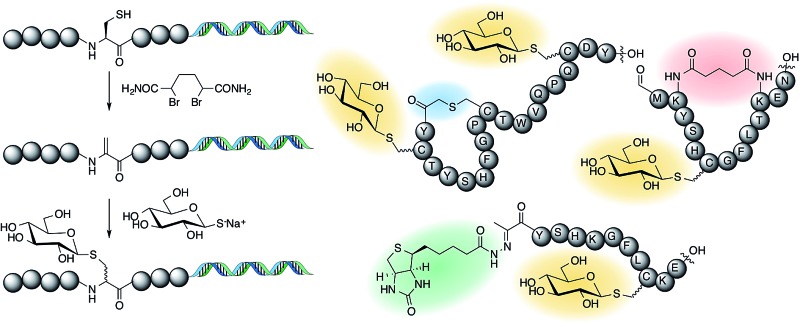
We report a strategy for efficient post-translational modification of a library of ribosomally-translated peptides by activation and elimination of cysteine to dehydroalanine then conjugate addition of a range of exogenous thiols, with an emphasis on carbohydrates.

## Introduction

Peptide drugs have recently been an area of great interest, driven in particular by their ability to modulate targets not receptive to traditional small-molecule therapeutics, such as protein–protein interactions, while retaining the potential for small-molecule-like pharmacokinetics.^1–4^ Carbohydrate-active enzymes and carbohydrate-binding proteins represent another area where peptide selection methods could make an important contribution, as these proteins have many important roles in many disease processes but can be difficult to target selectively and effectively with traditional therapeutics.^5^

*In vitro* ribosomal translation is able to very quickly and flexibly generate many different peptides, but it is generally limited to the chemical space of peptide linkages between the canonical amino acids. The available chemical space can be greatly expanded by the use of the flexible *in vitro* translation (‘FIT’) system^6^ – an integration of *in vitro* translation based on purified recombinant elements with ‘flexizymes’,^7–14^ which are a family of artificial ribozymes that are able to exogenously charge almost any amino acid onto *in vitro* transcribed tRNA, and thus greatly simplify genetic code reprogramming. While this system, as well as other genetic code reprogramming strategies^15,16^ can be used to introduce more varied functional groups than are present in the canonical amino acids, ribosomal incorporation of sterically demanding amino acid building blocks into a peptide chain remains as a bottleneck to increasing structural diversity. In particular, carbohydrate-modified amino acids appear to not be able to be translated efficiently (see Zhang *et al.*,^17^ and based on our own unpublished work), impeding the selection of novel glycopeptide lead compounds.

**Fig. 1:**
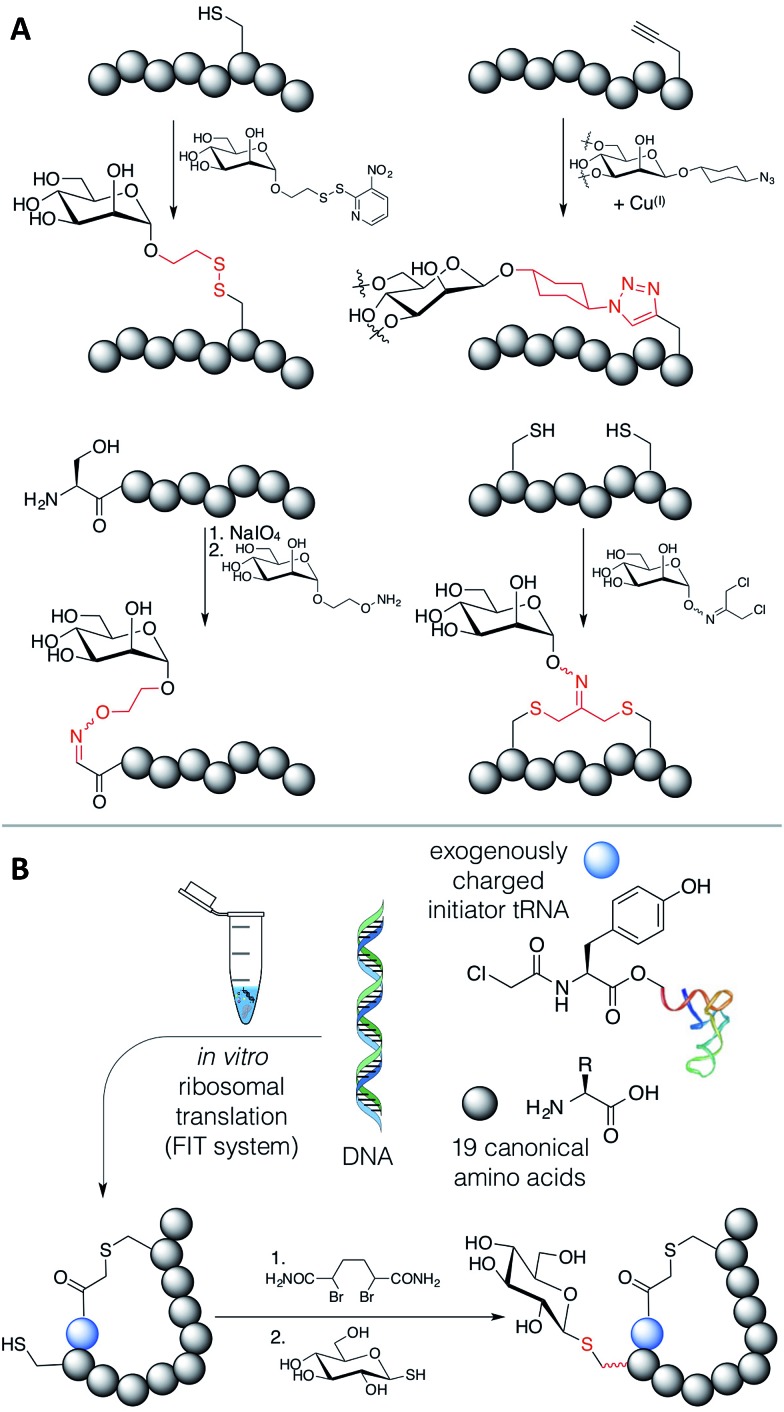
(A) Existing methods for post-translational incorporation of carbohydrates into a ribosomally-translated peptide library. Emphasised in red are the various linkers between the carbohydrates and peptides (see text for references). (B) Method presented in this work.

We have witnessed some methods that allow for the modification of ribosomally translated peptides with carbohydrates, including disulfide formation,^18^ azide–alkyne cycloaddition,^19^ sodium periodate oxidation of *N*-terminal serine residues followed by oxime formation,^20,21^ and alkylation of cysteines with dichloro-acetone followed by addition of a carbohydrate hydroxylamine^22^ or alkylation directly with the oxime derivative^23^ (Fig. 1A). However, a drawback of these methods is that the linkages generated are artificial; *i.e.* their structures significantly differ from the naturally occurring *N*- and *O*-glycosidic bonds.

An elegant alternative to these reactions is the conjugate addition of carbohydrate thiols to dehydroalanine (Dha), as shown in Fig. 1B, a methodology pioneered by the Davis group.^24^ This reaction gives rise to a thioacetal for carbohydrates, which has the advantage of closely approximating the natural linkage in *O*-glycosylation on serine. The thio-glycosidic linkage formed may be beneficial to provide resistance against enzymatic hydrolysis. We thus decided to initiate a program where this method was utilized to modify peptides, expressed in the FIT system, with carbohydrates.

Methods exist to install Dha^24,25^ (or dehydrobutyrine^25^) into a ribosomally translated peptide chain in an *in vitro* translation system, but we chose to apply the Davis group's bis-alkylating reagent, α,α′-dibromoadipic-bis-amide (DBAA), which activates a cysteine residue for elimination under mildly basic conditions.^26^ DBAA is highly selective for cysteines, easy to synthesise, and can be stably stored for extended periods. This method is readily applicable to the genetic code reprogramming of elongations codons with *N*-methyl-amino acids, for instance. DBAA has been extensively applied to studying post-translational modification of proteins, including ubiquitination,^27–29^ phosphorylation,^30–32^ glycosylation,^33^ and histone acetylation and methylation,^34^ to identification of catalytic residues,^35^ and to the display of chemically synthesized peptides including epitopes for antibody generation.^36–38^ It is not, however, obvious if this sequence of reactions is compatible with *in vitro* translation of peptides or with bioorthogonal reactions, due to the presence of many protein factors and enzymes, nucleotides and other small molecules, including reducing agents, β-mercaptoethanol (BME) and dithiothreitol (DTT). An investigation is thus presented here on the feasibility of DBAA-mediated elimination of cysteine followed by conjugate addition of an exogenous thiol to *in vitro* translated macrocyclic peptides generated by the FIT system, leading to a strategy to allow the *in vitro* selection of novel glycopeptide leads.

## Results and discussion

### Compatibility of the DBAA chemistry with the FIT system and macrocyclisation

As hoped, both the DBAA-mediated elimination and thiol conjugate addition reactions showed efficient and selective reactivity when applied to *in vitro* translated peptides. Conditions for these reactions were optimised first with purified peptide and subsequently in the FIT system without purification of the peptide product (Fig. S1–S5†). A short pre-reduction with DTT was included as a precaution against disulfide formation. As expected, negative controls with two further test peptides of arbitrary sequence (Fig. S6†), covering all 19 other canonical amino acids, showed no reactivity with DBAA, even under forcing conditions.

Macrocyclisation of peptides often leads to increased serum stability and increased target affinity.^39^ However, many cyclisation reactions for ribosomally translated peptides involve cysteine,^1^ and so are incompatible with DBAA. Despite this, some reactions remained promising for incorporation with the DBAA-mediated elimination and thiol conjugate addition strategy for carbohydrate incorporation. Reaction of an *N*-chloroacetylated initiator amino acid, incorporated by genetic code reprogramming using the FIT system,^40^ with a cysteine thiol is selective for the first downstream cysteine, with the important exception of a cysteine as the second amino acid.^41,42^ Cysteine in this second position could thus be used for modification within a macrocyclic peptide. In addition, a cysteine in the linear ‘tail’ outside the macrocyclic region could also be modified. Alternatively, the peptide could be cyclised with a cross-linking acylating agent such as disuccinimidyl glutarate (DSG), if the positioning and number of *N*-terminal- and lysine ε-amines is appropriate in the peptide.

**Fig. 2:**
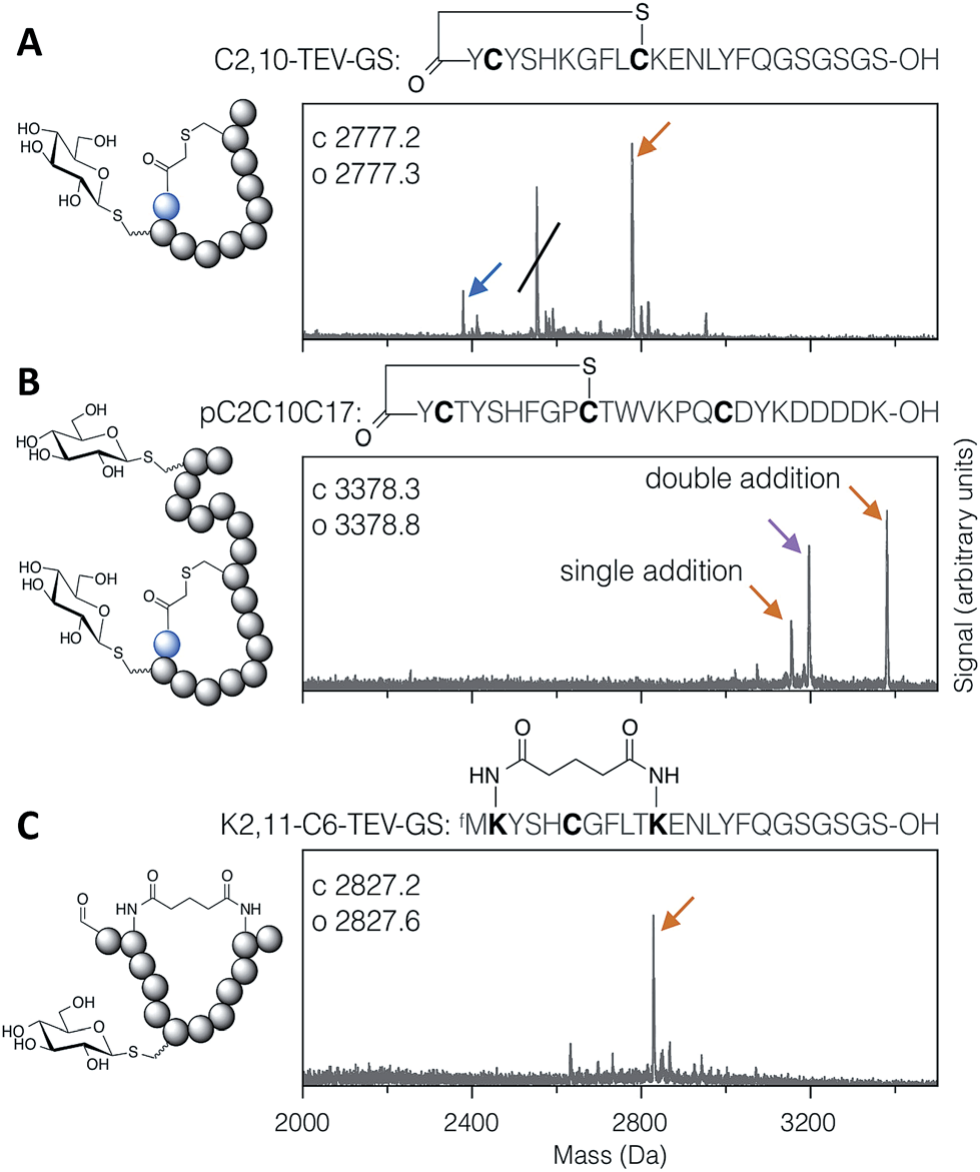
MALDI-TOF-MS spectra of example modified cyclic peptides. For each spectrum, the peptide sequence is shown above, with modified amino acids emphasised in bold, and the modifications are indicated in the cartoon structure to the left. (A) Thioether cyclised peptide with a single modification inside the macrocycle with thio-Glc. The struck-through peak is from a DBAA cross-linked side-product wherein translation started from the second codon. (B) Thioether cyclised peptide modified both inside and outside the macrocycle with thio-Glc. (C) Bis-amide cyclised peptide with a single modification inside the macrocycle with thio-Glc. Products are marked with colour-coded arrows as follows: blue for eliminated cysteine (Dha-containing peptide), orange for the conjugate addition product(s), and magenta for cysteines cross-linked with DBAA (see Fig. S7†). Calculated and observed values are for the final products, being the rightmost peak in each case.

Applying the DBAA-mediated elimination and thiol conjugate addition with 1-thio-β-d-glucose (as the sodium salt; ‘thio-Glc’) to two different thioether-macrocyclised peptides showed clean modification of a single cysteine at position 2 (Fig. 2A), as well as double modification of one peptide containing cysteines both inside and outside the macrocyclic portion (Fig. 2B). An additional peak is seen in both reactions arising from DBAA cross-linking of thiols (Fig. S7 and S8†), which in the case of the C2,10-TEV-GS peptide came from a second translation product starting at the second codon (and thus lacking the *N*-terminal chloroacetyl tyrosine). Similarly, macrocyclisation by DSG was shown to be compatible with DBAA-mediated elimination (Fig. 2C), provided that the macrocyclisation reaction was carried out before addition of excess thiol. Addition of DSG in one pot with the thiol resulted in reduced cyclisation efficiency, presumably because of reaction of the thiol with the succinimidyl ester (data not shown). Macrocyclic glycopeptides with both head-to-sidechain and sidechain-to-sidechain linkages were thus successfully synthesised using DBAA and thiol post-translational modification of ribosomally translated peptides.

### Substrate range for conjugate addition

**Fig. 3:**
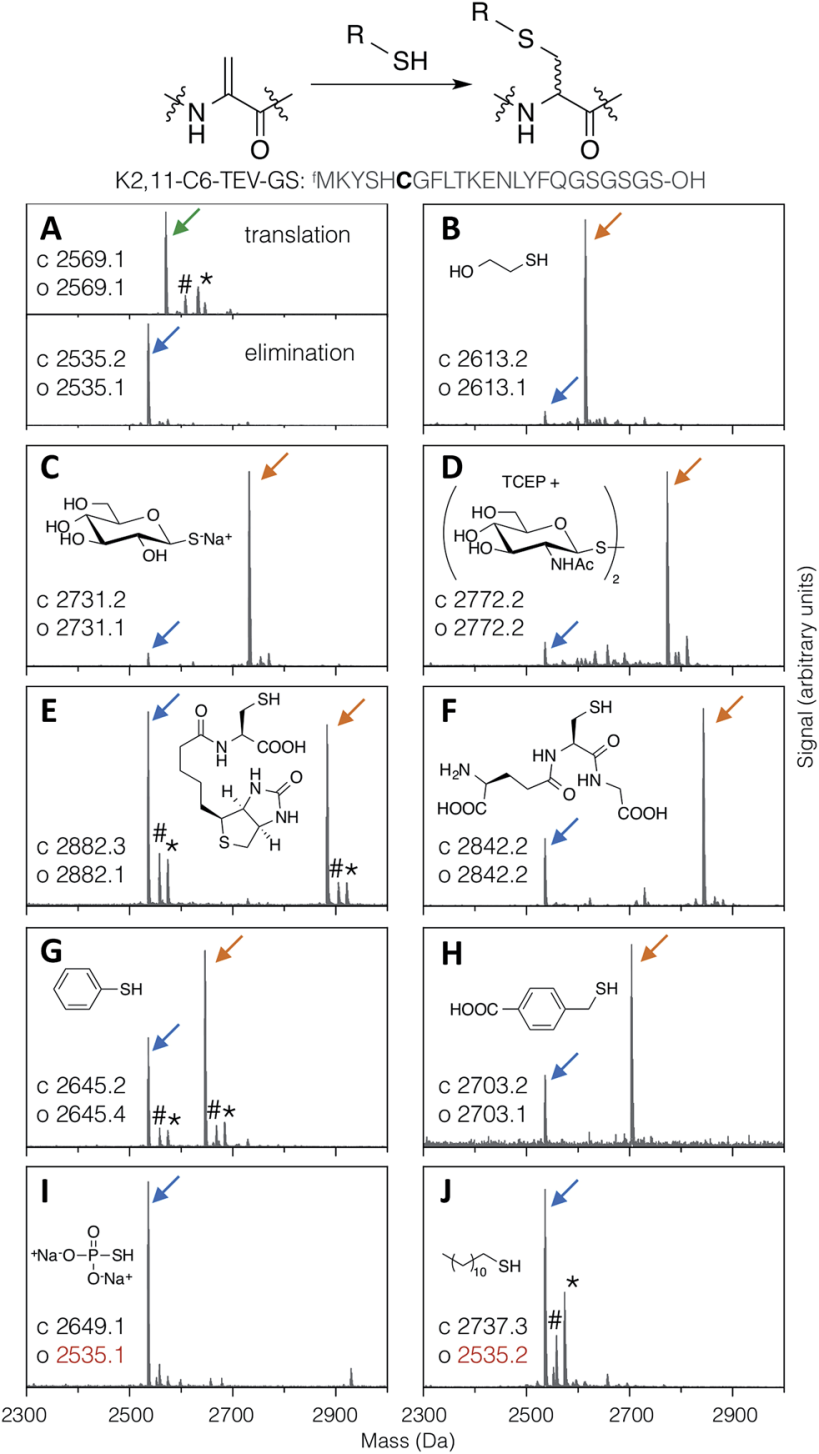
MALDI-TOF-MS spectra showing reaction efficiency for conjugate addition of various thiols (structures as shown) with the test peptide K2,11-C6-TEV-GS (sequence as shown, with site of modification emphasised in bold). Product peaks are indicated with colour-coded arrows as in Fig. 2, with the addition of green for the initial translation product (#: sodium adduct, *: potassium adduct).

Thiol addition to Dha has previously been employed to modify proteins with thiophosphate, thiol derivatives of carbohydrates, lipids, alkyl groups,^24^ and short peptides,^38^ as well as a more in depth study of alkyl, aryl and charged small thiols.^43^ The substrate scope for modification of *in vitro* translated peptides was anticipated to be similarly broad, and this was investigated by performing test additions using the following compounds: BME, thio-Glc, 1-thio-β-d-*N*-acetylglucosamine (as the disulfide, with pre-reduction; ‘thio-GlcNAc’), *N*-biotinylated cysteine, glutathione, thiophenol, 4-carboxybenzyl thiol, thiophosphate, and dodecane thiol (Fig. 3), which together exemplify alkyl, glycosidic, peptidic, aromatic, benzylic, phosphoryl, and lipidic thiols. Comparison with LC-ESI-MS data for the case of thio-Glc addition was used to validate the use of MALDI-TOF-MS to estimate progress of these reactions (Fig. S9†).

Most thiols tested showed acceptable reactivity with a Dha-containing test peptide (K2,11-C6-TEV-GS), with *N*-biotinyl cysteine being the slowest at around 50% conversion in 1.5 hours (Fig. 3E). These results are similar to those previously reported, where thiols close to a negative charge, *i.e.* carboxylate in this case, were found to give less efficient conversion.^43^ Surprisingly, we observed no reaction with thiophosphate (Fig. 3I), which is contrary to multiple reports in the literature.^30–32,34^ It is possible that some translation component is interfering with this reaction. Dodecanethiol also gave no reaction (Fig. 3J); since it was poorly water-soluble, resulting in partial precipitation during the reaction, the same effective concentration could perhaps not be achieved. Oxidised thiols, as disulfides, can also be used following a short pre-reduction with one equivalent of TCEP or DTT for 5 minutes at room temperature, as exemplified by the reaction with thio-GlcNAc (isolated as the disulfide form) pre-reduced by TCEP (Fig. 3D). Finally, multiple additions of thio-Glc to a model peptide were shown to be possible as long as sufficient thiol was added to overcome the high concentration of DBAA required to drive the elimination reaction (Fig. 2B for a macrocyclic peptide, data not shown for a linear peptide). These reactions illustrate the diversity of post-translational modifications that are possible with this strategy, in addition to the carbohydrates that are the focus of this report.

### Assessment of diastereoselectivity in the addition reaction

The conjugate addition of a thiol to Dha can give two stereo-outcomes, depending on the face from which a proton is added to the alpha carbon of the intermediate enolate (increasing to four stereoisomers for addition to dehydrobutyrine, also depending on the face from which nucleophilic attack occurs). This protonation step, which is the sole determinant of the stereochemical outcome for addition to Dha, occurs rapidly in aqueous media and is difficult to control. In peptides, this process has most thoroughly been studied for cyclisation of lanthipeptides, and can proceed with stereoselectivity in non-enzymatic reactions. This is particularly notable in the cyclisation of a Dha-Xaa-Xaa-Cys motif.^44–46^ Specificity arises from the effect of the peptide conformation on the local environment and the accessibility of the α-carbon to protons, under kinetic rather than thermodynamic control.^47^ This can have a large impact on the degree and nature of the selectivity, even in enzyme-mediated reactions.^48^

**Fig. 4:**
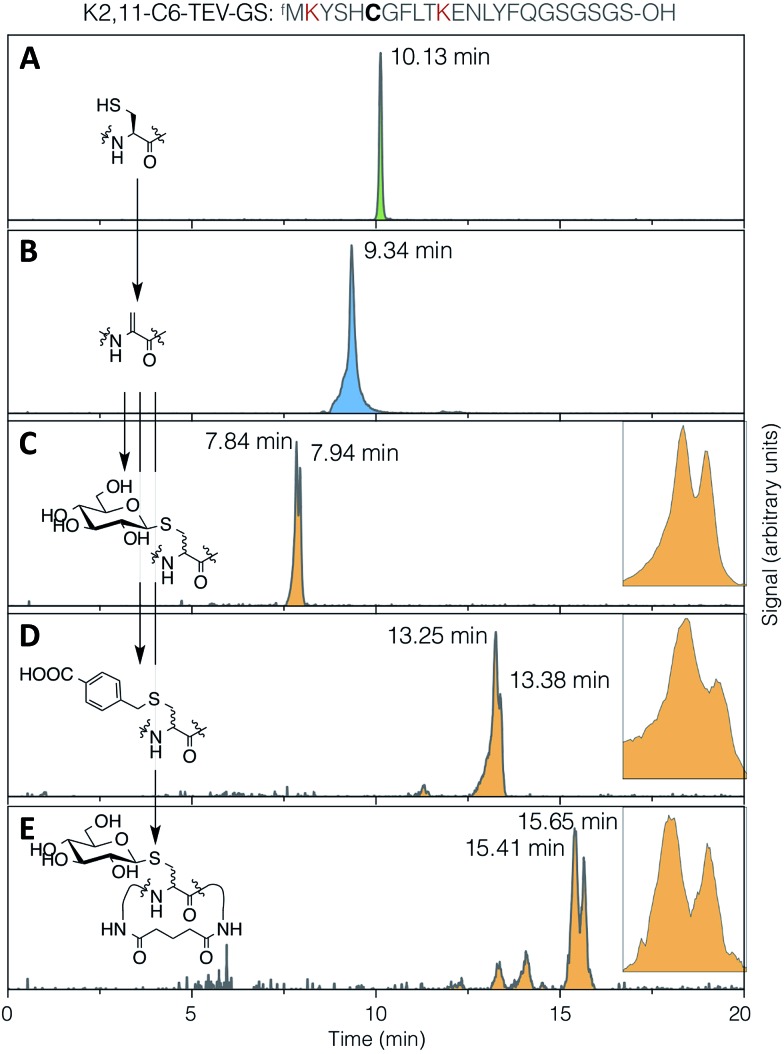
LC-ESI-MS traces for (A) translation, (B) elimination, (C) conjugate addition of 1-thio-β-d-glucose, (D) conjugate addition of 4-carboxybenzyl thiol, and (E) DSG cyclisation then conjugate addition of 1-thio-β-d-glucose, all with the test peptide K2,11-C6-TEV-GS (sequence as shown at top, with cysteine emphasised in bold and the lysines used for cyclisation in red). Traces are extracted ion chromatograms for the relevant [M + 2H]^2+^ species (1285.0–1286.5, 1268.0–1269.5, 1352.0–1353.5, 1366.0–1367.5, 1414.0–1415.5, respectively; TIC traces in ESI Fig. S10†). Inset: expansions of the partially-resolved peaks for conjugate addition product diastereomers.

The stereoselectivity of exogenous thiol addition to Dha-containing ribosomally translated peptides was investigated using LC-ESI-MS. The translated unmodified peptide was found to elute as a single peak at 10.13 min (Fig. 4A), the Dha-containing peptide as a single peak at 9.34 min (Fig. 4B), and the products of reaction with thio-Glc as two partially resolved peaks at 7.84 and 7.94 min (Fig. 4C), in a diastereomeric ratio of approximately 65 : 35 (as estimated by peak area). Similarly, the products of addition of 4-carboxybenzyl thiol manifested as two partially resolved peaks at 13.25 min and 13.38 min (Fig. 4D), in a diastereomeric ratio of 75 : 25, showing some influence from the nature of the nucleophile. Which of these peaks corresponds to which isomer cannot be determined from mass data alone, but this does demonstrate that the reaction proceeds with more specificity than initially expected. The diastereoselectivity observed is not sufficient to be able to know *a priori* which isomer is dominant, but this is not prohibitive for the use of the reactions presented here in peptide discovery. It should be noted that a fresh stock of thio-Glc was used each time for these reactions, in order to prevent complications from anomerisation (which was confirmed by NMR to not occur in 3 hours). For the case of a less abundant thiol, such as a synthetic oligosaccharide rather than commercially available monosaccharide, a similar effect to preparing a fresh stock can be achieved by storing as a disulfide, thus preventing anomerisation, and reducing immediately before use with a stoichiometric amount of DTT or TCEP.

It might be expected that peptide conformational restriction by macrocyclisation would improve this diastereoselectivity, as any induced secondary structure may mean the two faces of the alpha carbon in the intermediate enolate have different solvent accessibility. However, addition of thiol to the same peptide as above macrocyclised by DSG (Fig. 4E) gave a diastereomeric ratio of approximately 55 : 45, indicating that this is not true, at least for this particular sequence. Several other smaller peaks were also observed for this product, possibly arising from slowly interconverting minor conformers. The same pattern of multiple peaks was also present for the DSG-macrocyclised peptide without added thiol (Fig. S10†), confirming that these do not arise from further changes in stereochemistry, such as anomerisation. A similar level of diastereoselectivity to our observations described above was also reported for addition of a peptide thiol to Dha in ubiquitin, in either native or denaturing conditions and at two different locations,^28^ corroborating the modest stereochemical control observed here.

Most importantly, we did not observe mass peaks in the LC-ESI-MS data that correspond to anticipated possible side products, including DTT, BME, or cysteine forming conjugate adducts with Dha, disulfides with peptidyl cysteine, or mixed adducts with DBAA. This further corroborates the MALDI-TOF-MS analyses. Even though an amount of such side products undetectable by the LC-ESI-MS analysis could be formed, the trace level of such contaminants is unlikely to interfere with further discovery processes using an appropriate *in vitro* display system.

### *N*-Terminal modification

During optimisation and characterisation of the DBAA-mediated elimination reaction it was observed that DBAA can also be used for the creation of an *N*-terminal ketone. Reaction of an *N*-terminal cysteine with Dha affords an enamine, and this initially-formed enamine undergoes spontaneous tautomerisation to an imine, followed by hydrolysis to a ketone. This ketone can be used for further derivatisation of the peptide, for example with a hydrazine or hydroxylamine. This allows for selective functionalisation of the *N*-terminal ketone and an internal Dha with different molecules in a single peptide, and so provides a route for display of two different groups – for example two different carbohydrates, or a carbohydrate and an affinity tag or fluorophore (Fig. S11†).

### Compatibility with the RaPID system

In order to demonstrate the suitability of the reaction sequence presented here for peptide discovery, the compatibility of DBAA-mediated elimination and thiol conjugate addition was subsequently also tested with the RaPID system (for ‘Random non-standard Peptides Integrated Discovery’).^6,11,13,49–54^ This system is a combination of FIT translation and our optimised version of cDNA-stabilised mRNA display, for *de novo* discovery of peptides containing non-canonical building blocks. To complete the selection process, it is required to show not only the selective modification of peptides with thio-Glc but also the subsequent PCR amplification of cDNA without perturbation.

**Fig. 5:**
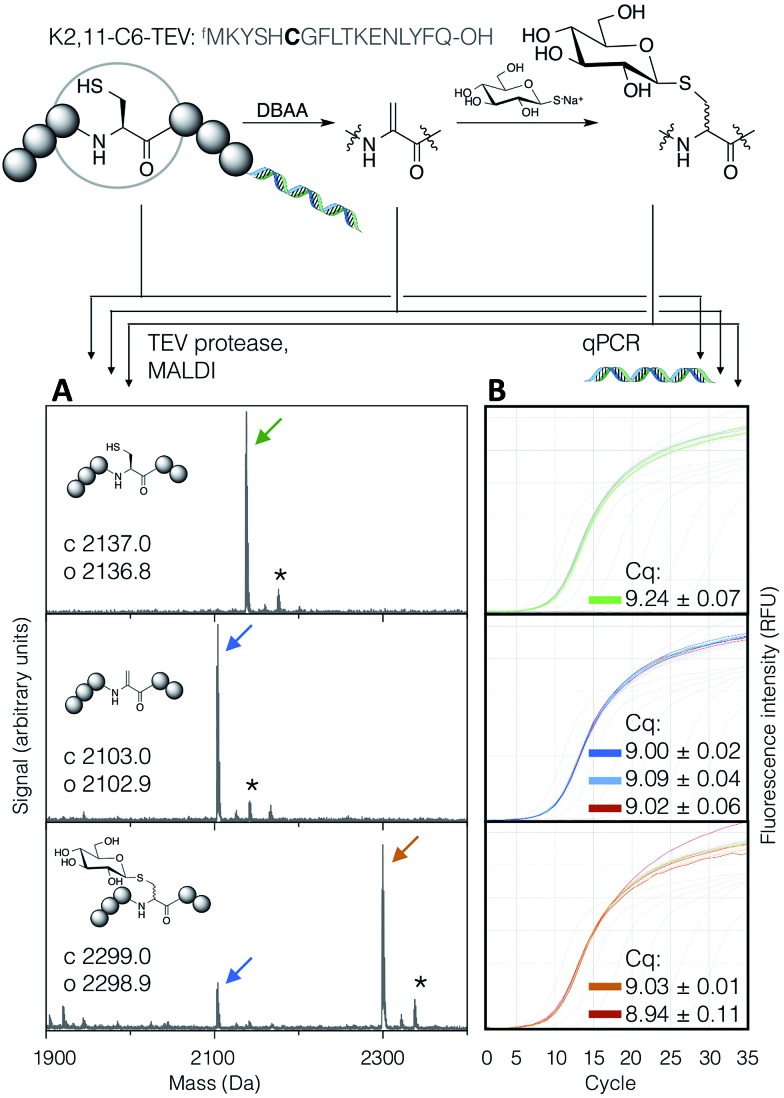
Compatibility of the DBAA-mediated elimination and thiol conjugate addition reactions with an mRNA displayed test peptide (sequence shown at top, with site of modification emphasised in bold). On the left are MALDI-TOF-MS spectra for the peptides at each stage of modification, following cleavage off the mRNA by TEV protease, while on the right are qPCR traces for amplification of the cDNA from the same step. Cq values are averages of triplicate measurements ± standard error. Traces and peak markers are colour-coded by product as in Fig. 2, with the addition of green for the unmodified translation product, red for solvent-only negative controls in the qPCR amplification plots, and dark blue for a higher concentration of DBAA (*: potassium adduct).

To test this, a model peptide (K2,11-C6-TEV-GS) was synthesised as its mRNA/cDNA conjugate, reacted with DBAA then thio-Glc, and the integrity of the peptide and nucleic acid components verified. This peptide contains a TEV protease recognition sequence, which allows the peptide to be selectively cleaved off the mRNA tag under mild conditions for direct analysis of the displayed molecule by MALDI-TOF-MS. Both modification reactions showed satisfactory conversion (Fig. 5A). Furthermore, the amplification of cDNA from modified and unmodified peptides, as well as solvent-only negative controls for each step, were compared by qPCR (Fig. 5B), and all were found to be equivalent. This demonstrates that peptide modification by the above reactions is efficient at the concentration range of mRNA-displayed peptides (low micromolar) in the presence of the *in vitro* translation system, and that the reactions do not affect the nucleic acid component of the system. Thus the strategy reported here should be suited to incorporation with peptide-display based discovery platforms, allowing rapid identification of bioactive *de novo* glycopeptides.

## Conclusion

Glycoproteins are important biomolecules, involved in many cellular recognition and immune processes. In order to accelerate the discovery of molecules that can modulate these processes, a strategy has been developed to allow the incorporation of diverse carbohydrates in a ribosomally translated macrocyclic peptide scaffold. In this strategy, reaction of peptidyl cysteines with DBAA followed by addition of exogenous thiols gives a thioglycosidic connection that closely mimics natural *O*-glycosylation on serine. These reactions were also shown to be compatible with two different peptide macrocyclisation strategies, and allow functionalisation with two different compounds on the *N*-terminus and internally. These reactions neither damage nucleic acids nor interfere with cDNA amplification by PCR, meaning they are compatible with the RaPID system or analogous mRNA display techniques. Because the modification is purely chemical, it can be readily applicable to various kinds of carbohydrate thiol and peptide sequence.

## Experimental section

### Materials and reagents

Unless otherwise specified, reagents were purchased from Sigma-Aldrich, Tokyo Chemical Industry co. (TCI) or Wako Pure Chemical Industries, and used as-supplied without further purification. Oligonucleotides were purchased from Eurofins Genomics as dry pellets following OPC purification (sequences in Table S1†), and DNA templates for *in vitro* translation were assembled by PCR and isolated by precipitation with 70% ethanol in 0.3 M NaCl. MALDI-TOF-MS spectra were recorded on a Bruker Autoflex or UltrafleXtreme system following desalting by C-Tip (AMR Inc.) and elution with 50% saturated α-cyano-4-hydroxycinnamic acid in aqueous 80% MeCN, 0.5% acetic acid. Literature procedures were used for synthesis of DBAA,^26^ *N*-biotinyl cysteine (using biotin-NHS instead of sulfo-NHS),^55^ di-(*N*-acetyl-β-d-glucosaminyl)-disulfide,^56^ and 4-(mercaptomethyl)benzoic acid.^57^

### Aminoacylation of tRNA by flexizyme

Exogenously charged amino-acyl tRNA were prepared using enhanced flexizyme (eFx) as previously reported.^12^ Briefly, eFx and tRNAfMetCAU (25 μM each) in HEPES–KOH buffer (50 mM, pH 7.5) were thermally denatured, then cooled to room temperature and allowed to fold in the presence of MgCl_2_ (600 mM). Cyanomethyl ester-activated *N*-chloroacetyl-l-tyrosine in DMSO (5 mM final) was then added to the pre-chilled solution and allowed to react on ice for 2 hours before quenching with four volumes of 0.3 M NaOAc (pH 5.2) and precipitation by addition of ethanol to 70%. Pellets were stringently washed, then stored dry at –80 °C.

### *In vitro* translation and genetic code reprogramming

Peptides were translated using the FIT system,^12^ with genetic code reprogramming by omission of canonical amino acids and replacement with exogenously-charged amino-acyl tRNA. Translation reactions were carried out at 37 °C for 30 minutes. The components of the translation system are as follows: 1.2 μM ribosome, 0.1 μM T7 RNA polymerase, 4 μg mL^–1^ creatine kinase, 3 μg mL^–1^ myokinase, 0.1 μM pyrophosphatase, 0.1 μM nucleotide-diphosphatase kinase, 0.73 μM AlaRS, 0.02 μM CysRS, 0.13 μM AspRS, 0.23 μM GluRS, 0.68 μM PheRS, 0.09 μM GlyRS, 0.02 μM HisRS, 0.4 μM IleRS, 0.11 μM LysRS, 0.04 μM LeuRS, 0.03 μM MetRS, 0.38 μM AsnRS, 0.16 μM ProRS, 0.06 μM GlnRS, 0.03 μM ArgRS, 0.04 μM SerRS, 0.09 μM ThrRS, 0.02 μM ValRS, 0.03 μM TrpRS, 0.02 μM TyrRS, 0.26 μM EF-G, 10 μM EF-Tu, 10 μM EF-Ts, 2.7 μM IF1, 0.4 μM IF2, 1.5 μM IF3, 0.6 μM MTF, 0.25 μM RF2, 0.17 μM RF3, 0.5 μM RRF, 1.5 mg mL^–1^ *Escherichia coli* total tRNA, 0.5 mM of all 20 proteinogenic amino acids (except for methionine, as outlined below), 2 mM ATP, 1 mM UTP, 2 mM GTP, 1 mM CTP, 2 mM spermidine, 20 mM creatine phosphate, 2 mM DTT, 50 mM HEPES–KOH (pH 7.6), 12 mM magnesium acetate, 100 mM potassium acetate. Translation was initiated by addition of template DNA to a final concentration of 100 nM, unless otherwise stated. For templates with a TAG stop codon, RF1 was also included at 0.25 μM.

For non-reprogrammed translation, 10-formyltetrahydrofolate (1 mM) was included along with all 20 canonical amino acids.

For peptides initiated with *N*-chloroacetyl-l-tyrosine, methionine was omitted and aminoacylated initiator tRNAfMetCAU was included at 12.5 μM.

For translation of the C2,10-TEV-GS test peptide with an *N*-terminal cysteine, methionine was omitted and not replaced.

### General method for elimination and thiol addition to peptides

Following *in vitro* translation, sodium phosphate buffer pH 8.5 (40 mM) and DTT (0.5 mM) were added and the reaction incubated at 42 °C for 5 minutes. A pre-warmed stock solution of DBAA at 10 times the final concentration in DMF (50 or 500 mM, for one or multiple Cys per peptide, respectively) was then added and the elimination reaction allowed to proceed at 42 °C. In the case of 50 mM final concentration of DBAA, a small amount of precipitate was typically seen by the end of the reaction, but did not appear to decrease elimination efficiency (this precipitate was presumed to be either DBAA or 2,5-dicarboxamidothiophane; the cyclised product following elimination from cysteine). Temperature of DBAA addition is important, as addition of a high concentration of DBAA to a cold solution immediately gave rise to a large amount of precipitation. For the conjugate addition reaction, thiol in either water or DMSO was added (in excess over DBAA) and the reaction allowed to proceed at 37 °C for at least 1 hour.

### Macrocyclisation of peptides

For cyclisation by a head-to-sidechain thioether linkage in the pC2C10C17 and C2,10-TEV-GS test peptides, translation was initiated with *N*-chloroacetyl-l-tyrosine as outlined above and modified using the above general methods for elimination (5 and 50 mM DBAA, respectively) and addition of 1-thio-β-d-glucose (50 mM thiol, 1.5 hours).

For cyclisation by cross-linking of lysine side chain amines in the K2,11-C6-TEV-GS test peptide, 0.8 mM DSG (Thermo Fisher Scientific) was added after the elimination reaction (5 mM DBAA), and allowed to react at 37 °C for 30 min. This was repeated for a total of 3 additions, followed by modification with 1-thio-β-d-glucose using the above general method (12.5 mM, 3 hours).

### Peptide analysis by LC-ESI-MS

LC-ESI-MS analysis was carried out as published.^42^ Briefly, 20 μL translation reactions were subjected to the above general method for post-translational modification of peptides with 5 mM DBAA then 12.5 mM thio-Glc or carboxybenzyl thiol for 3 hours. Macrocyclisation by DSG was also carried out as outlined above. Following confirmation of the reaction product by MALDI-TOF-MS using 5 μL, the remaining 15 μL were then diluted with 45 μL 50% MeCN in water with 0.1% formic acid, and any debris pelleted by a brief benchtop centrifugation. From this, 10 μL was injected onto a Thermo Accela liquid chromatograph interfaced with a Thermo Exactive Orbitrap mass spectrometer. Separation was achieved by a Phenomenex Aeris Peptide column (XB-C18, 150 mm × 2.1 mm, dp = 3.6 μm) using a 5 minute initial gradient from 100% to 80% solvent A (5% acetonitrile, 10 mM ammonium formate, 10 mM formic acid, aq.) in solvent B (95% acetonitrile, 10 mM ammonium formate, 10 mM formic acid, aq.), followed by a 15 minute gradient from 80% to 75% solvent A in solvent B, at a flow rate of 600 μL min^–1^ for both gradient sections.

### Compatibility with the RaPID system

An mRNA-displayed version of the K2,11-C6-TEV-GS test peptide was prepared as previously described,^58^ with *in vitro* translation started by addition of puromycin-ligated mRNA (1.2 μM) at 70 μL scale. Ribosomes were disrupted by addition of 10 mM EDTA, and the mRNA reverse transcribed for one hour at 42 °C using the GS3an-2.R36 primer. The reaction (cumulative volume 160 μL) was then split in half, with one half used for modification and the other half for a negative control. Elimination (5 and 50 mM DBAA, two separate reactions) and conjugate addition (11.2 mM 1-thio-β-d-glucose following 5 mM DBAA, 1.5 hour incubation) were carried out following the above general methods, with DMF then water used for the negative controls under otherwise identical conditions. Samples were taken after reverse transcription, elimination, and conjugate addition (20, 22.2 and 25 μL, respectively; each equivalent to 1/4 of the total initial volume) and diluted with water to 25 μL. From this, 1 μL was diluted to 500 μL with water then analysed by qPCR, while the remainder was desalted into Tris–HCl–EDTA buffer pH 8.0 (50 and 0.5 mM, respectively) by passing through pre-equilibrated sephadex G-25 gel filtration resin. Treatment with AcTEV protease (5 units, Thermo Fisher Scientific) for 2 hours at 25 °C without added reducing agent gave the free *N*-terminal fragment of the peptide (^f^MKYSHCGFLTKENLYFQ-OH), which was analysed by MALDI-TOF-MS. RT-qPCR was carried out using a Lightcycler Nano (Roche) with SYBR green dye, the primers T7g10M.F46 and stop-an2.R19, running 35 cycles of 95 °C for 10 s, 61 °C for 10 s, 72 °C for 30 s.

## References

[cit1] Passioura T., Katoh T., Goto Y., Suga H. (2014). Annu. Rev. Biochem..

[cit2] Giordanetto F., Kihlberg J. (2014). J. Med. Chem..

[cit3] Bhat A., Roberts L. R., Dwyer J. J. (2015). Eur. J. Med. Chem..

[cit4] Craik D. J., Fairlie D. P., Liras S., Price D. (2013). Chem. Biol. Drug Des..

[cit5] Hart G. W., Copeland R. J. (2010). Cell.

[cit6] Hipolito C. J., Suga H. (2012). Curr. Opin. Chem. Biol..

[cit7] Murakami H., Saito H., Suga H. (2003). Chem. Biol..

[cit8] Murakami H., Ohta A., Ashigai H., Suga H. (2006). Nat. Methods.

[cit9] Xiao H., Murakami H., Suga H., Ferré-D'Amaré A. R. (2008). Nature.

[cit10] Ohuchi M., Murakami H., Suga H. (2007). Curr. Opin. Chem. Biol..

[cit11] Passioura T., Suga H. (2013). Chem.–Eur. J..

[cit12] Goto Y., Katoh T., Suga H. (2011). Nat. Protoc..

[cit13] Passioura T. and Suga H., in Topics in current chemistry, Springer, 2014, vol. 344, pp. 331–345. 10.1007/128_2013_421 23478876

[cit14] Morimoto J., Hayashi Y., Iwasaki K., Suga H. (2011). Acc. Chem. Res..

[cit15] Chin J. W. (2014). Annu. Rev. Biochem..

[cit16] Hartman M. C. T., Josephson K., Lin C.-W., Szostak J. W. (2007). PLoS One.

[cit17] Zhang Z., Gildersleeve J., Yang Y.-Y., Xu R., Loo J. A., Uryu S., Wong C.-H., Schultz P. G. (2004). Science.

[cit18] Arai K., Tsutsumi H., Mihara H. (2013). Bioorg. Med. Chem. Lett..

[cit19] Horiya S., Bailey J. K., Temme J. S., Guillen Schlippe Y. V., Krauss I. J. (2014). J. Am. Chem. Soc..

[cit20] Ng S., Lin E., Kitov P. I., Tjhung K. F., Gerlits O. O., Deng L., Kasper B., Sood A., Paschal B. M., Zhang P., Ling C.-C., Klassen J. S., Noren C. J., Mahal L. K., Woods R. J., Coates L., Derda R. (2015). J. Am. Chem. Soc..

[cit21] Kitov P. I., Vinals D. F., Ng S., Tjhung K. F., Derda R. (2014). J. Am. Chem. Soc..

[cit22] Assem N., Ferreira D. J., Wolan D. W., Dawson P. E. (2015). Angew. Chem., Int. Ed..

[cit23] Ng S., Derda R. (2016). Org. Biomol. Chem..

[cit24] Bernardes G. J. L., Chalker J. M., Errey J. C., Davis B. G. (2008). J. Am. Chem. Soc..

[cit25] Goto Y., Iwasaki K., Torikai K., Murakami H., Suga H. (2009). Chem. Commun..

[cit26] Chalker J. M., Gunnoo S. B., Boutureira O., Gerstberger S. C., Fernández-González M., Bernardes G. J. L., Griffin L., Hailu H., Schofield C. J., Davis B. G. (2011). Chem. Sci..

[cit27] Haj-yahya N., Hemantha H. P., Meledin R., Bondalapati S., Mallikanti S., Brik A. (2014). Org. Lett..

[cit28] Meledin R., Mali S. M., Singh S. K., Brik A. (2016). Org. Biomol. Chem..

[cit29] Mulder M. P. C., El Oualid F., Ter Beek J., Ovaa H. (2014). ChemBioChem.

[cit30] Rowan F. C., Richards M., Bibby R. A., Thompson A., Bayliss R., Blagg J. (2013). ACS Chem. Biol..

[cit31] Chooi K. P., Galan S., Raj R., Mccullagh J. S. O., Mohammed S., Jones L. H., Davis B. G. (2014). J. Am. Chem. Soc..

[cit32] Gunnoo S. B., Finney H. M., Baker T. S., Lawson A. D., Anthony D. C., Davis B. G. (2014). Nat. Commun..

[cit33] Lercher L., Raj R., Patel N. A., Price J., Mohammed S., Robinson C. V., Schofield C. J., Davis B. G. (2015). Nat. Commun..

[cit34] Chalker J. M., Lercher L., Rose N. R., Schofield C. J., Davis B. G. (2012). Angew. Chem., Int. Ed..

[cit35] Timms N., Windle C. L., Polyakova A., Ault J. R., Trinh C. H., Pearson A. R., Nelson A., Berry A. (2013). ChemBioChem.

[cit36] Morrison P. M., Foley P. J., Warriner S. L., Webb M. E. (2015). Chem. Commun..

[cit37] Kaur H., Harris P. W. R., Little P. J., Brimble M. A. (2015). Org. Lett..

[cit38] Vallée M. R. J., Schombs M. W., Balaban Z. J., Colyer J., Davis B. G. (2016). Chem. Commun..

[cit39] Mallinson J. (2012). Future Med. Chem..

[cit40] Goto Y., Ohta A., Sako Y., Yamagishi Y., Murakami H., Suga H. (2008). ACS Chem. Biol..

[cit41] Iwasaki K., Goto Y., Katoh T., Suga H. (2012). Org. Biomol. Chem..

[cit42] Bashiruddin N. K., Nagano M., Suga H. (2015). Bioorg. Chem..

[cit43] Rowan F., Richards M., Widya M., Bayliss R., Blagg J. (2014). PLoS One.

[cit44] Zhou H., van der Donk W. A. (2002). Org. Lett..

[cit45] Okeley N. M., Zhu Y., van Der Donk W. A. (2000). Org. Lett..

[cit46] Toogood P. L. (1993). Tetrahedron Lett..

[cit47] Zhu Y., Gieselman M. D., Zhou H., Averin O., van der Donk W. A. (2003). Org. Biomol. Chem..

[cit48] Tang W., Jiménez-Osés G., Houk K. N., van der Donk W. A. (2015). Nat. Chem..

[cit49] Ito K., Passioura T., Suga H. (2013). Molecules.

[cit50] Morioka T., Loik N. D., Hipolito C. J., Goto Y., Suga H. (2015). Curr. Opin. Chem. Biol..

[cit51] Jongkees S. A. K., Hipolito C. J., Rogers J. M., Suga H. (2015). New J. Chem..

[cit52] Rogers J. M., Suga H. (2015). Org. Biomol. Chem..

[cit53] Terasaka N., Iwane Y., Geiermann A.-S., Goto Y., Suga H. (2015). Int. J. Mol. Sci..

[cit54] Bashiruddin N. K., Suga H. (2015). Curr. Opin. Chem. Biol..

[cit55] Li W. W., Gong L., Bayley H. (2013). Angew. Chem., Int. Ed..

[cit56] Kiefel M., Thomson R., Radovanovic M., von Itzstein M. (1999). J. Carbohydr. Chem..

[cit57] Niwa N., Yamagishi Y., Murakami H., Suga H. (2009). Bioorg. Med. Chem. Lett..

[cit58] Yamagishi Y., Shoji I., Miyagawa S., Kawakami T., Katoh T., Goto Y., Suga H. (2011). Chem. Biol..

