# Supplementary material for: Linker-free incorporation of carbohydrates into in vitro displayed macrocyclic peptides
Source: Chem Sci. 2016 Oct 21;8(2):1474–81. doi: 10.1039/c6sc04381j (PMC5452274; doi:10.1039/c6sc04381j)
Supplement: Supplementary file 1 [file SC-008-C6SC04381J-s001.pdf]

Electronic Supplementary Material (ESI) for Chemical Science.
This journal is © The Royal Society of Chemistry 2016

Supplementary materials for:

Linker-free incorporation of glycosides into *in vitro* displayed macrocyclic peptides

S. A. K. Jongkees,[a] S. Umemoto[a] and H. Suga[a,b]

[a] *Department of Chemistry, Graduate School of Science, The University of Tokyo, 7-3-1 Hongo, Bunkyo-ku, 113-0033 Tokyo, Japan.*

[b] *JST CREST, The University of Tokyo, 7-3-1 Hongo, Bunkyo-ku, 113-0033 Tokyo, Japan.*

Supplementary methods 2
Primers and DNA template assembly 3
Initial optimisation of DBAA elimination and thiol conjugate addition reactions 4
MALDI spectra for optimisation of DBAA conditions with SPPS peptide 5
MALDI spectra for optimisation of DBAA conditions with translated peptide........ 6
MALDI spectra for optimisation of conditions for thiol conjugate addition 9
MALDI spectra for DBAA reaction negative controls........ 10
MALDI spectra for effect of DBAA concentration on product ratio 12
Comparison of MALDI-TOF-MS and LC-ESI-MS analysis of reaction progress........ 13
TIC and supplementary XIC traces for LC-ESI-MS data........ 14
Supplementary references........ 16

Supplementary methods

Assembly of DNA templates for in vitro translation

DNA templates were assembled as follows:

- Pep3 was selected from a library of random sequences, isolated by TA-cloning (unpublished data, coding sequence shown in Table S1), and further amplified with T7g10M.F48 and GS3an-2.R36 primers by PCR. For the purposes of this work, it was used only as a convenient on-hand macrocyclic test peptide containing a free cysteine.
- pC17 through pC2C10C17 (Fig. S7) were assembled as previously reported.[1]
- K2,11-C6-TEV-GS was assembled by the same method as pC17 through pC2C10C17, using the primers K2-C6-TEVext.R49 for the extension reaction and C6-TEV-GSPCR.R49 for the first PCR reaction.
- C2,10-TEV-GS was assembled by extension using the above-listed primers of the same name, followed by PCR using T7g10M.F46 and GS3an-2.R36.
- DBAAneg1 and DBAAneg2 were assembled by a single extension reaction using T7g10M.F48 and the above primers of the same name.

Optimisation of elimination and addition reactions

For initial testing of the elimination reaction, HPLC-purified test peptide pep3 (25 μM) was dissolved in buffer as listed and DBAA added from a stock at 20 times final concentration in DMSO. The reaction was allowed to proceed at the temperature and for the time indicated, then desalted and analysed by MALDI-TOF-MS. For optimisation of the conjugate addition reaction, the general method for elimination was used (5 mM DBAA), followed by addition of 4-carboxybenzyl thiol from a stock at 10 times the final concentration in DMSO. The reaction was again allowed to proceed at the temperature and for the time indicated, then desalted and analysed by MALDI-TOF-MS. Reactions with *in vitro* translated peptides were optimised in the same manner, varying reaction time, temperature, and reagent concentration.

Negative controls were carried out based on the general method for elimination (45 mM DBAA, 2 hour incubation) with peptides DBAAneg1 (^{f}MKTYHFGPLTWPQKG-OH) and DBAAneg2 (^{f}MKARSWRVNDGIEKG-OH).

pep3 synthesis

The test peptide pep3 was synthesised using standard Fmoc SPPS chemistry with a *C*-terminal $(PEG)_5$ linker and *N*-terminally capped by chloroacetyl-NHS, followed by solution phase cyclisation and RP-HPLC purification.[2] The N-terminal amino acid of synthetic pep3 was *N*-chloroacetyl-D-tryptophan, but when translated ribosomally *N*-chloroacetyl-L-tyrosine was used as initiator amino acid, for convenience only.

Modification of N-terminal cysteines

For *N*-terminal biotinylation of a dehydroalanine-derived ketone, the C2,10-TEV-GS test peptide was translated in the absence of an initiating amino acid, and reduced by 5 mM DTT for 10 minutes at 42 °C. Following elimination (50 mM DBAA) and addition of 1-thio-β-D-glucose (42.8 mM thiol, 3 hours) using the above general methods, biotin hydrazide (13.75 mM) and sodium acetate pH 5.2 (150 mM) were added and the reaction allowed to proceed at 37 °C for 3 hours.

Primers and DNA template assembly

Table S1: Primer and cds sequences for templates used

Primer/cds	Sequence
Pep3 cds	ATGGCGGCTGATGATATTTGGTATGGTCGTGTTTGTCTCTGCGGCAGCGGCAGCGGCAGCTAG
T7g10M.F46	TAATACGACTCACTATAGGGTTAACTTTAAGAAGGAGATATACATA
T7g10M.F48	TAATACGACTCACTATAGGGTTAACTTTAAGAAGGAGATATACATATG
GS3an-2.R36	TTTCCGCCCCCCGTCCTAGCTGCCGCTGCCGCTACC
Stop-an2.R19	TTTCCGCCCCCCGTCCTAG
K2-C6-TEVext.R49	CTTTGGTCAGGAAACCGCAGTGAGAGTATTTCATATGTATATCTCCTTC
C6-TEV-GSPCR.R49	GCCGCTGCCGCTACCCTGGAAGTACAGGTTTTCTTTGGTCAGGAAACCG
C2,10-TEV-GS.F55	CTTTAAGAAGGAGATATACATATGTGCTATAGCCATAAAGGCTTTCTGTGCAAAG
C2,10-TEV-GS.R54	GCCGCTGCCGCTACCCTGAAAATACAGGTTTTCTTTGCACAGAAAGCCTTTATG
DBAAneg1.R70	GCTTCGTTACCCCTTCTGAGGCCAGGTCAGCGGGCCAAAATGATAGGTCTTCATATGTATATCTCCTTCT
DBAAneg2.R70	GCTTCGTTAACCCTTCTCAATACCATCATTCACCCGCCAACTACGAGCCTTCATATGTATATCTCCTTCT

Initial optimisation of DBAA elimination and thiol conjugate addition reactions

Initial optimisation of conditions for DBAA-mediated conversion of cysteine to dehydroalanine was carried out with an HPLC-purified macrocyclic peptide synthesised by standard Fmoc chemistry ('pep3'; ClAc-^{D}WAADDIWYGRVCLCGSGSGS-$(PEG)_5$-NH_2, cyclised from the *N*-terminal chloroacetate to the first cysteine as a thioether, as shown on the next page), with product detection by MALDI-TOF-MS. A survey of initial conditions (Fig. S1) indicated that pH 8.5 was sufficiently basic for DBAA-mediated elimination of 25 µM peptidyl cysteine thiol by 2.5 mM DBAA at 37 °C for one hour, and phosphate buffer gave faster reaction than HEPES or Tris buffer of similar pH. Applying these conditions to a ribosomally-translated peptide of the same sequence (initiated with L-Tyr instead of D-Trp, for convenience) also lead to elimination, but reaction was incomplete. This is likely the result of competing reactions between DBAA and the components of the *in vitro* translation mix, such as proteins and free thiols. Further optimisation was thus carried out, varying temperature, DBAA concentration, and reaction time (Fig. S2-4). Final optimised conditions were 5 mM DBAA at 42 °C for one hour to give good conversion, but this incubation was extended to 1.5 hours to ensure complete conversion of any recalcitrant peptide sequences that may be present in a library of random peptides. Negative controls for the DBAA reaction were carried out using two peptides of arbitrary sequence, covering all amino acids except cysteine between them. No reaction was observed with either peptide in 45 mM DBAA at 42 °C for 2 hours (Fig. S6).

Having established suitable conditions for the introduction of dehydroalanine into a ribosomally-translated peptide, conditions for the subsequent conjugate addition of a thiol were also investigated. An initial test with β-mercaptoethanol showed that addition of thiol in excess over DBAA to the crude reaction mixture gave conjugate addition, meaning intermediate purification is not required (data not shown). Optimisation was subsequently carried out using 4-carboxybenzyl thiol as a convenient on-hand representative post-translational modification. Conjugate addition was found to be slower than DBAA-mediated elimination, with little effect from temperature or thiol concentration in the range tested (Fig. S5). One hour appeared to be sufficient for the majority of the peptide to react, and overnight incubation gave complete conversion (data not shown). For the application of this reaction to peptide selections, the degree of conversion seen in one hour is acceptable, while the incubation time could be extended if complete conversion is required. As with the elimination reaction, a slightly longer incubation was adopted as standard to allow for recalcitrant sequences in a library of random peptides. Final optimised conditions for the conjugate addition reaction were thus 10 mM thiol at 37 °C for 1.5 hours in pH 8.5 phosphate buffer.

MALDI spectra for optimisation of DBAA conditions with SPPS peptide

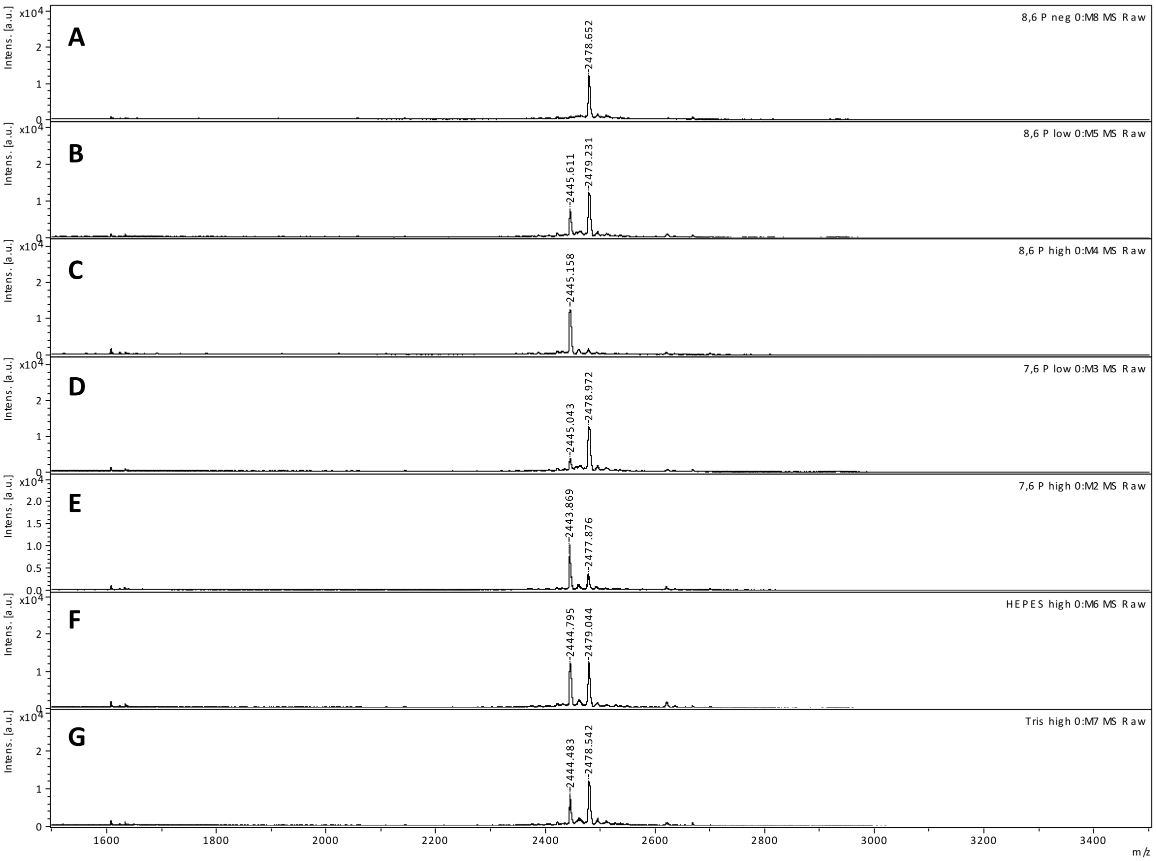

Fig. S1 MALDI-TOF-MS spectra from a screen of initial conditions for DBAA reaction with solid-phase synthesised pep3. (A) negative control, 40 mM phosphate buffer pH 8.6 only (B) 0.25 mM DBAA, 40 mM phosphate buffer pH 8.6 (C) 2.5 mM DBAA, 40 mM phosphate buffer pH 8.6 (D) 0.25 mM DBAA, 40 mM phosphate buffer pH 7.6 (E) 2.5 mM DBAA, 40 mM phosphate buffer pH 7.6 (F) 2.5 mM DBAA, 40 mM Tris buffer pH 8.0 (G) 2.5 mM DBAA, 40 mM HEPES buffer pH 7.5, all reacted for 1 hour at 37 °C. Calculated mass for synthetic mono-isotopic pep3: starting material (Cys-containing) 2477.1, product (Dha-containing) 2443.1

pep3 from SPPS:

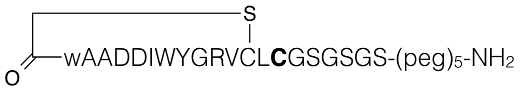

MALDI spectra for optimisation of DBAA conditions with translated peptide

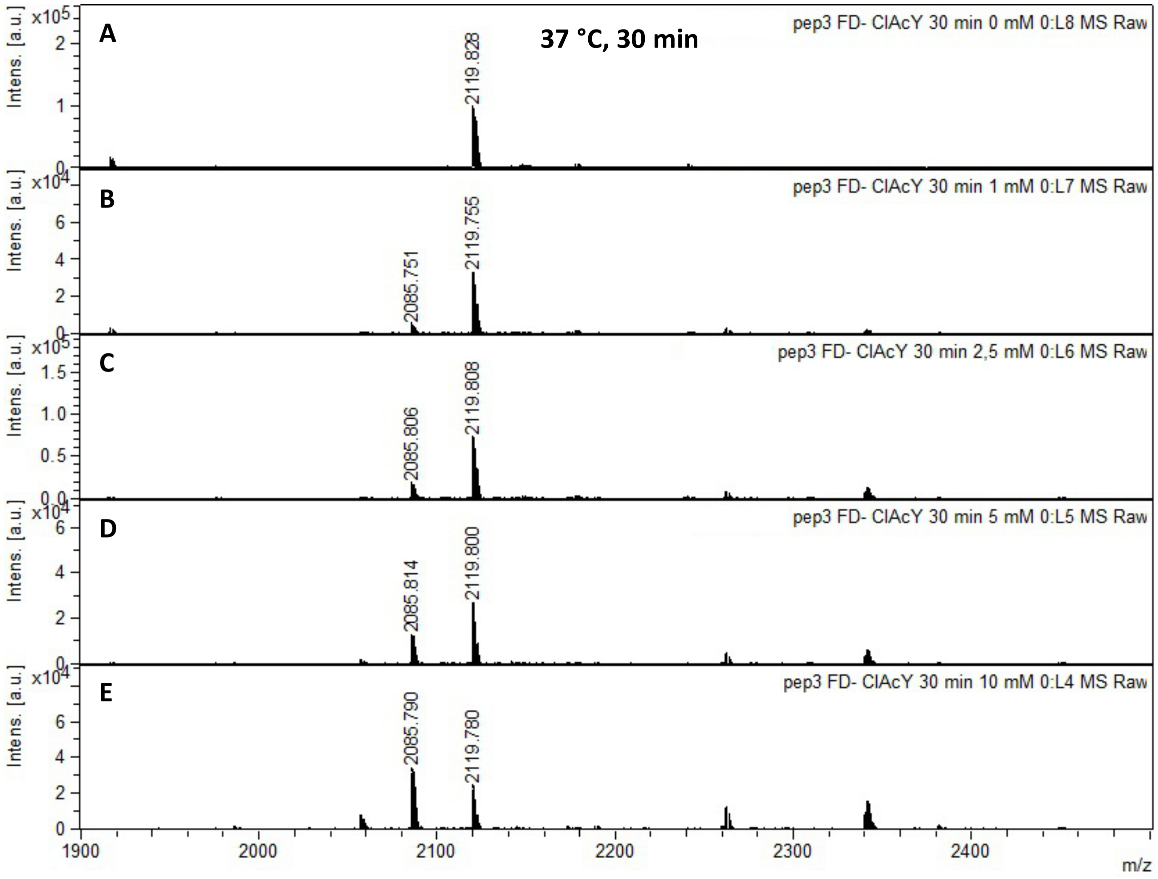

Fig. S2 MALDI-TOF-MS spectra from optimisation of conditions for DBAA reaction with ribosomally translated pep3, all at 37 °C for 30 min. (A) negative control (DMSO only) (B) 1 mM DBAA (C) 2.5 mM DBAA (D) 5 mM DBAA (E) 10 mM DBAA. Calculated mass for mono-isotopic ribosomally-translated pep3: starting material 2119.9, DBAA treated 2085.9, with additional transient peaks at 2339.9 and 2261.0 Da from the DBAA linear and cyclised adducts, respectively (not marked).

pep3 from ribosomal translation:

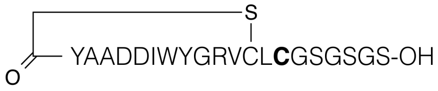

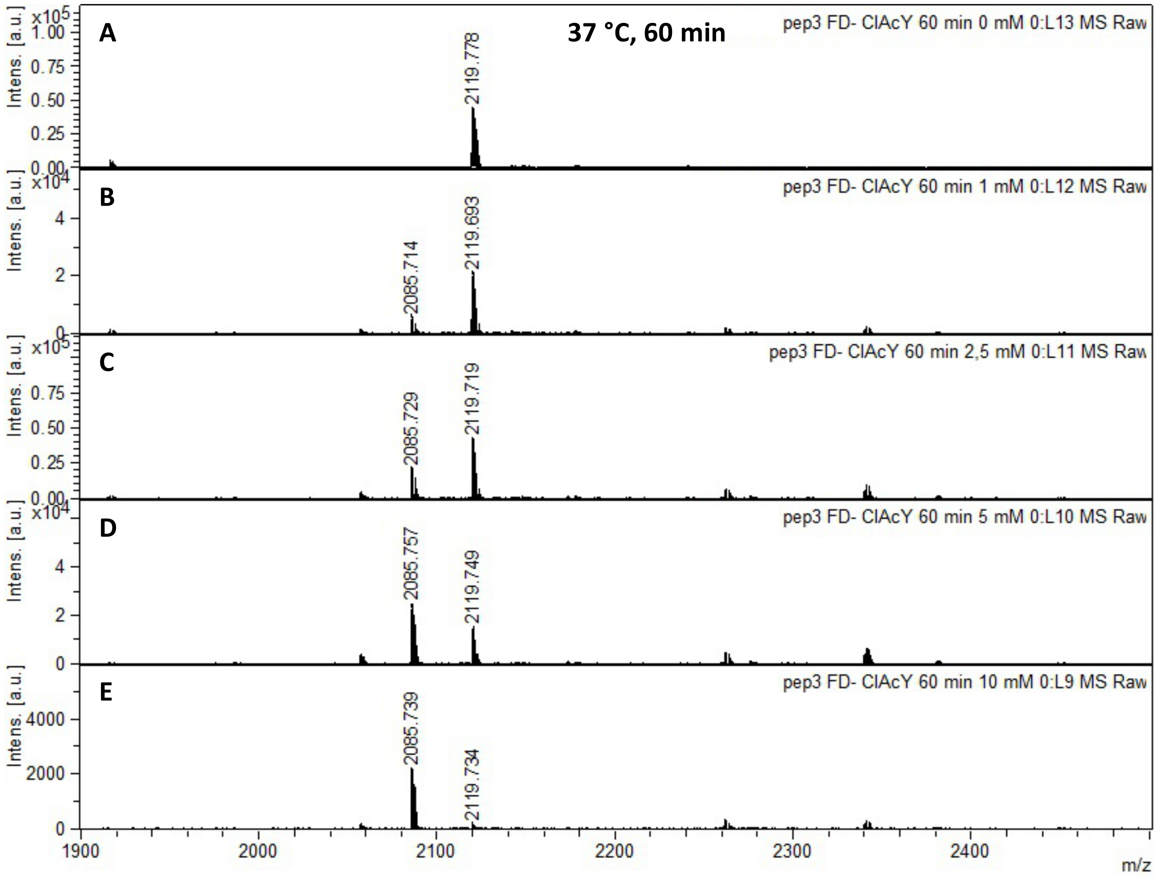

Fig. S3 MALDI-TOF-MS spectra from optimisation of conditions for DBAA reaction with ribosomally translated pep3, all at 37 °C for 60 min. (A) negative control (DMSO only) (B) 1 mM DBAA (C) 2.5 mM DBAA (D) 5 mM DBAA (E) 10 mM DBAA. Peptide masses and structures as in Fig. S2.

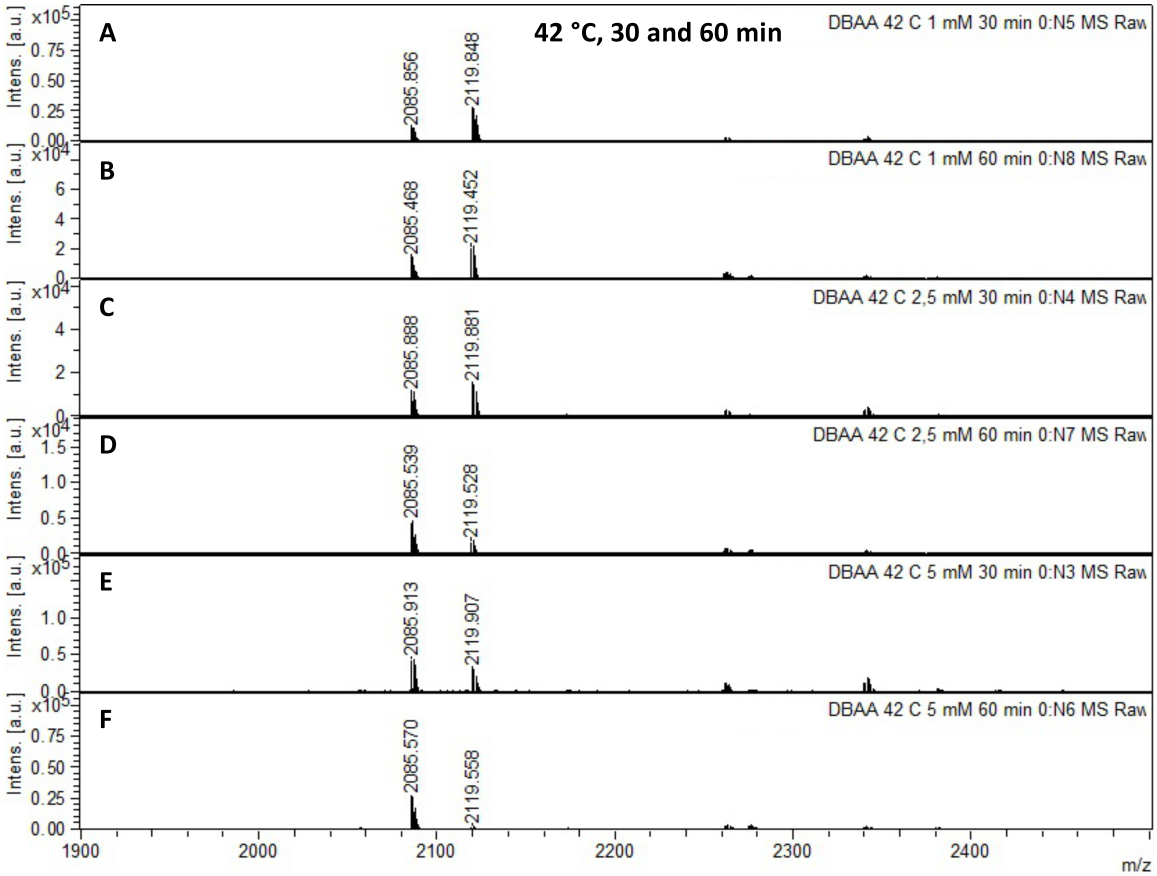

Fig. S4 MALDI-TOF-MS spectra from optimisation of conditions for DBAA reaction with ribosomally translated pep3, all at 42 °C. (A) 1 mM DBAA for 30 min (B) 1 mM DBAA for 60 min (C) 2.5 mM DBAA for 30 min (D) 2.5 mM DBAA for 60 min (E) 5 mM DBAA for 30 min (F) 5 mM DBAA for 60 min. Peptide masses and structures as in Fig. S2.

MALDI spectra for optimisation of conditions for thiol conjugate addition

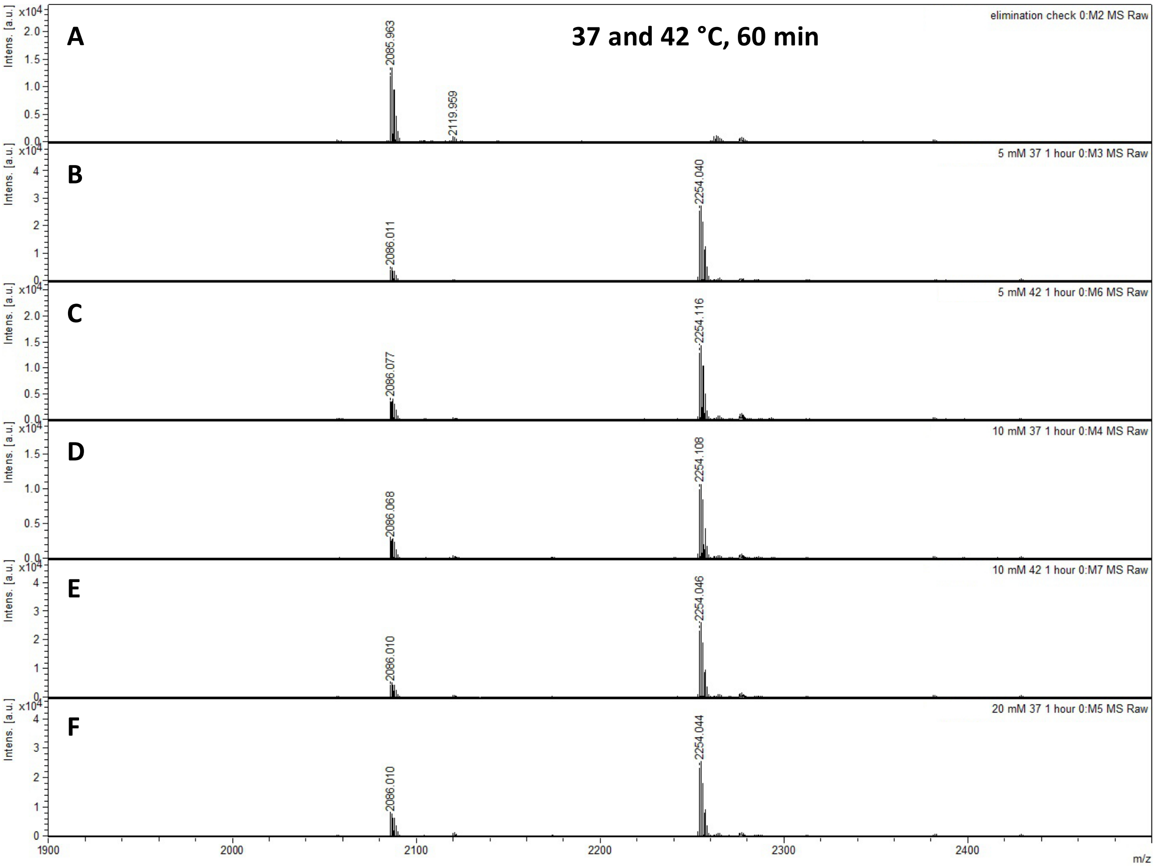

Fig. S5 MALDI-TOF-MS spectra from optimisation of conditions for conjugate addition of 4-carboxybenzyl thiol to dehydroalanine in ribosomally translated pep3, all for 60 min. (A) elimination check, 5 mM DBAA under optimised conditions (B) 5 mM thiol, 37 °C (C) 5 mM thiol, 42 °C (D) 10 mM thiol, 37 °C (E) 10 mM thiol, 42 °C (F) 20 mM thiol, 37 °C. Calculated mass for mono-isotopic ribosomally-translated pep3: starting material 2119.9, DBAA treated 2085.9, thiol addition 2253.9.

MALDI spectra for DBAA reaction negative controls

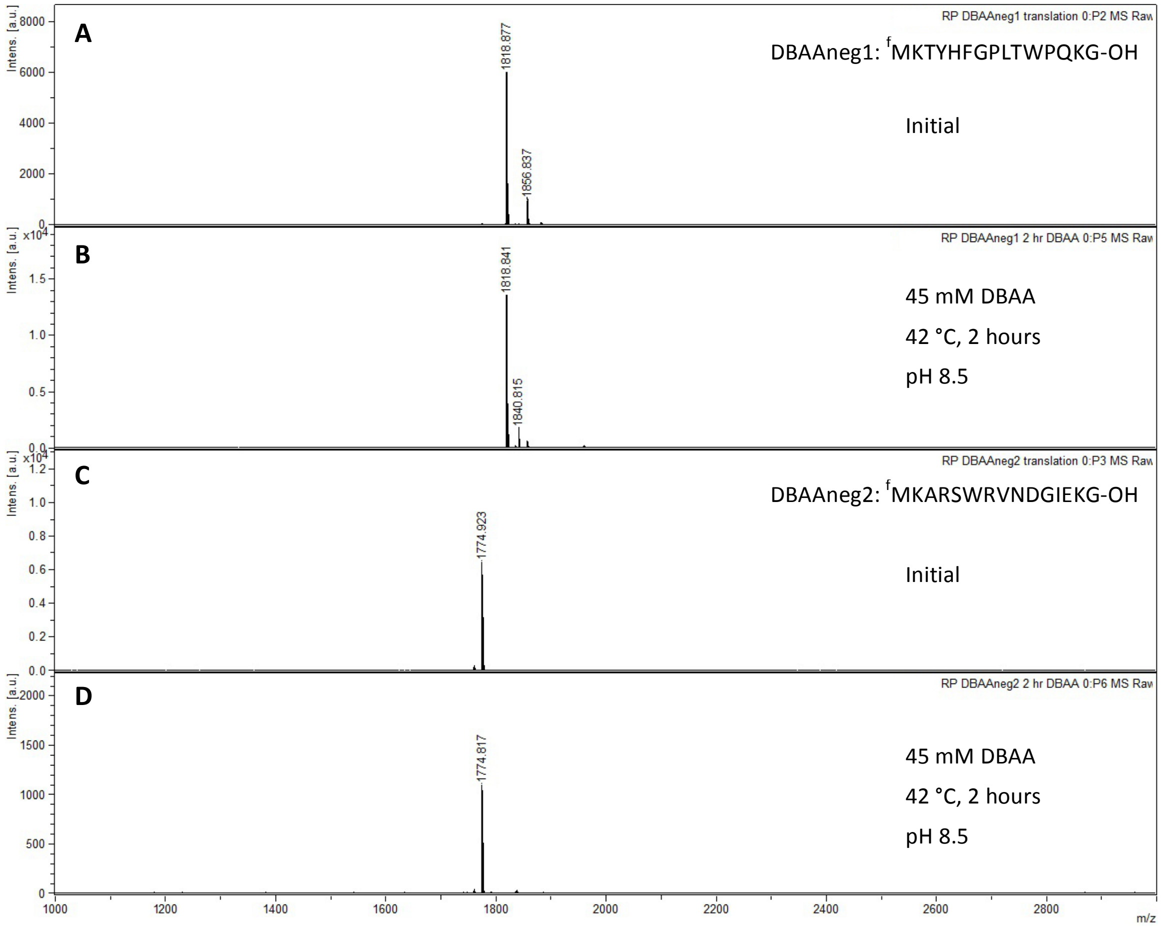

Fig. S6 MALDI-TOF-MS spectra for DBAA negative controls with two different peptides, covering all 19 canonical amino acids except cysteine. (A) translation of DBAAneg1 (B) treatment of DBAAneg1 with 45 mM DBAA for 2 hours at 42 °C (C) translation of DBAAneg2 (D) treatment of DBAAneg2 with 45 mM DBAA for 2 hours at 42 °C. Calculated mass for mono-isotopic ribosomally-translated DBAAneg: 11818.9, for DBAAneg2: 1774.9.

Effect of multiple cysteines

For selection of glycopeptide modulators of biologically relevant interactions, multivalent display may be of great benefit. The DBAA-mediated elimination and thiol conjugate addition reactions were thus applied to a set of ribosomally-translated peptides with multiple cysteines at varied sequence distances.[1]

Conversion of a single cysteine proceeded smoothly with the above-optimised conditions for DBAA-mediated elimination (5 mM reagent). However, peptides with multiple cysteines showed predominantly cross-linking, rather than elimination, with the remainder of the product of a mass consistent with a single elimination (Fig. S7). This was hypothesised to be a lanthionine cross-link, which was supported by a lack of reaction with added exogenous thiol (data not shown). Cross-linking of multiple thiols in one peptide by DBAA has also been reported by Morrison *et al.*,[3] which they solved through use of a bis-alkylating reagent that has a reduced rate for the second alkylation relative to the first. It was anticipated that a similar effect could be achieved by increasing the concentration of DBAA, accelerating the intermolecular first alkylation relative to the intramolecular subsequent reaction. Indeed, increasing the concentration of DBAA to 20 mM gave around 50% of the doubly eliminated product, while at 40 mM the proportion of cross-linked product decreased substantially (Fig. S8). Also shown in Fig. S7 are the products arising from treatment of the same set of test peptides with 50 mM DBAA, which all gave satisfactory conversion. Only the closest cysteines showed an increase in the proportion of cross-linking under these conditions, but double elimination remained the main product. However, applying these conditions to peptides containing three cysteines continued to give a mixture of cross-linked peptides as the major product (with the third cysteine eliminating to dehydroalanine). Two dehydroalanine residues can thus be created in a single peptide using 50 mM DBAA at 42 °C for one hour, but more would require use of an alternate reagent or method.

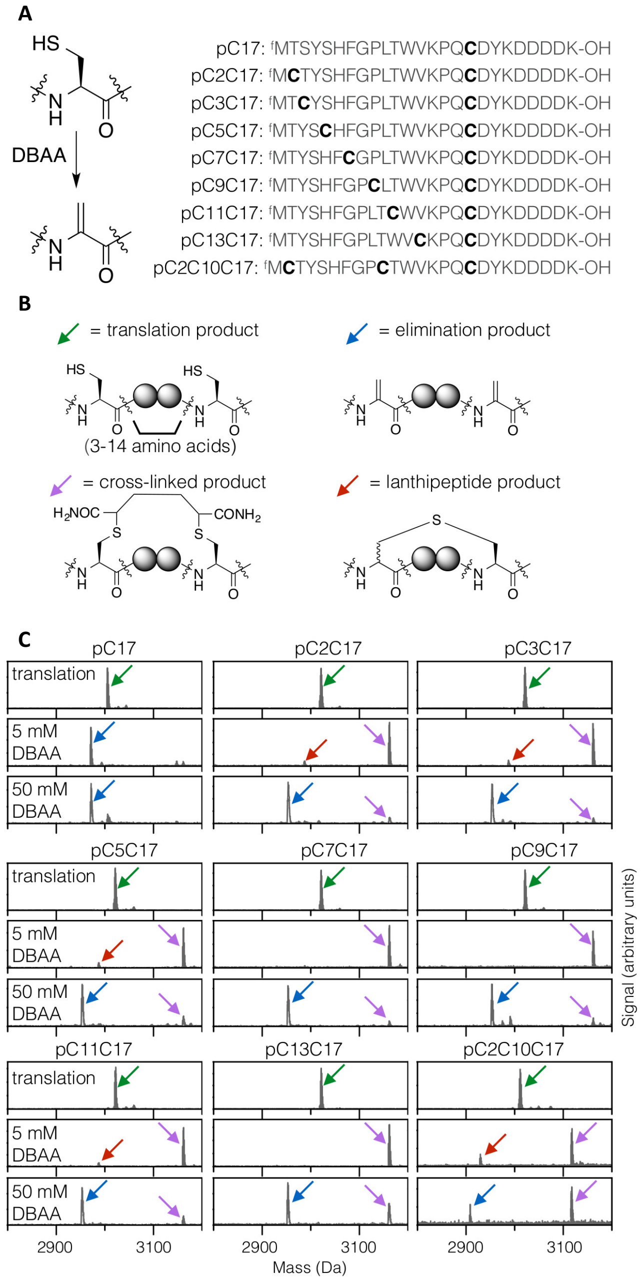

Fig. S7 Reaction of DBAA with peptides containing multiple cysteines. (A). Desired reaction and test peptide sequences, with cysteines emphasised in bold. (B). Possible products for reaction of DBAA with peptides containing multiple cysteines. (C). MALDI-TOF-MS spectra for the peptides in A before and after being subjected to either 5 or 50 mM DBAA for 1.5 hours in pH 8.5 phosphate buffer at 42 °C. Products are indicated with colour-coded arrows as outlined in B.

MALDI spectra for effect of DBAA concentration on product ratio

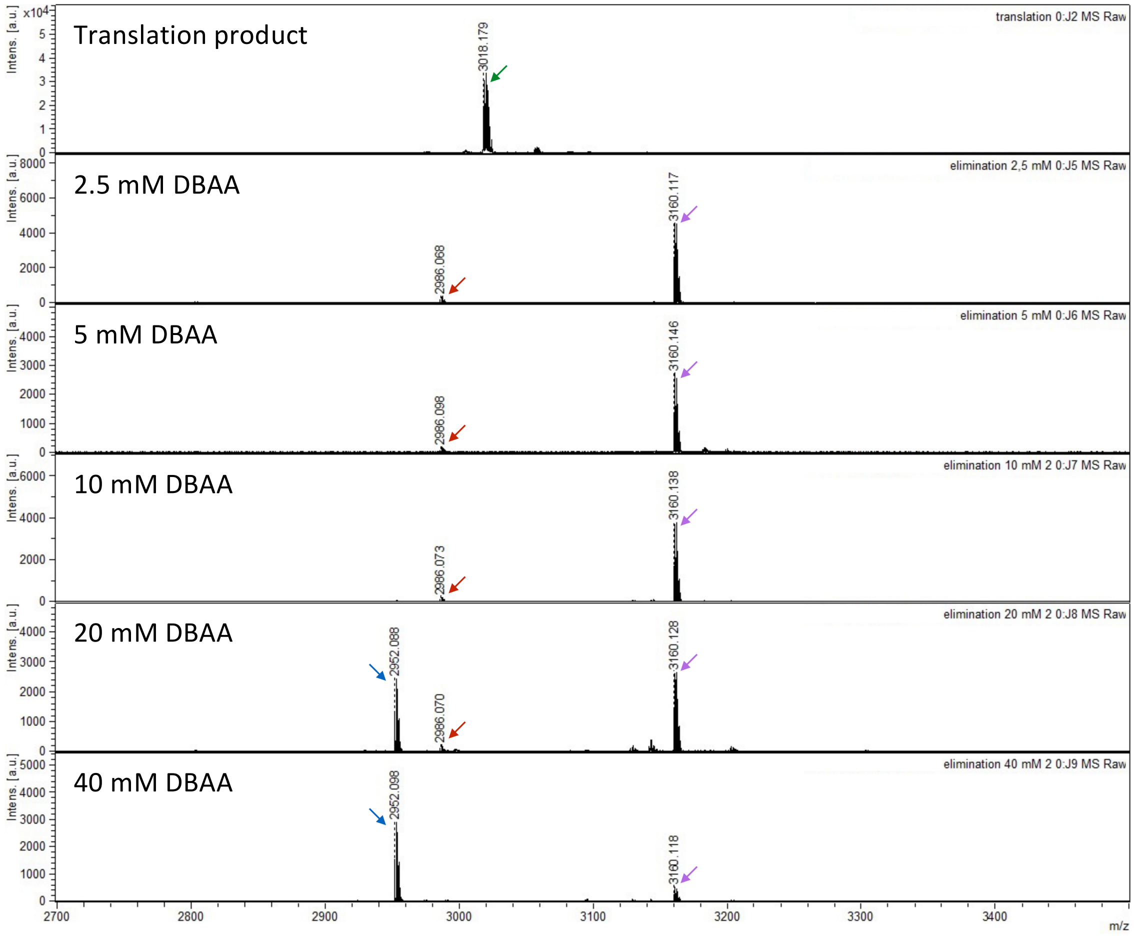

Fig. S8 MALDI-TOF-MS spectra showing the effect of increasing DBAA concentration on the relative abundance of different products in the pC2C17 test peptide (Fig. S7). Calculated mass for mono-isotopic pC2C17: starting material 3018.3 (as intramolecular disulfide), peptide with two dehydroalanines 2952.3, DBAA cross-linked peptide 3160.4, lanthipeptide 2986.3, with colour coding as in Fig. S7.

Comparison of MALDI-TOF-MS and LC-ESI-MS analysis of reaction progress

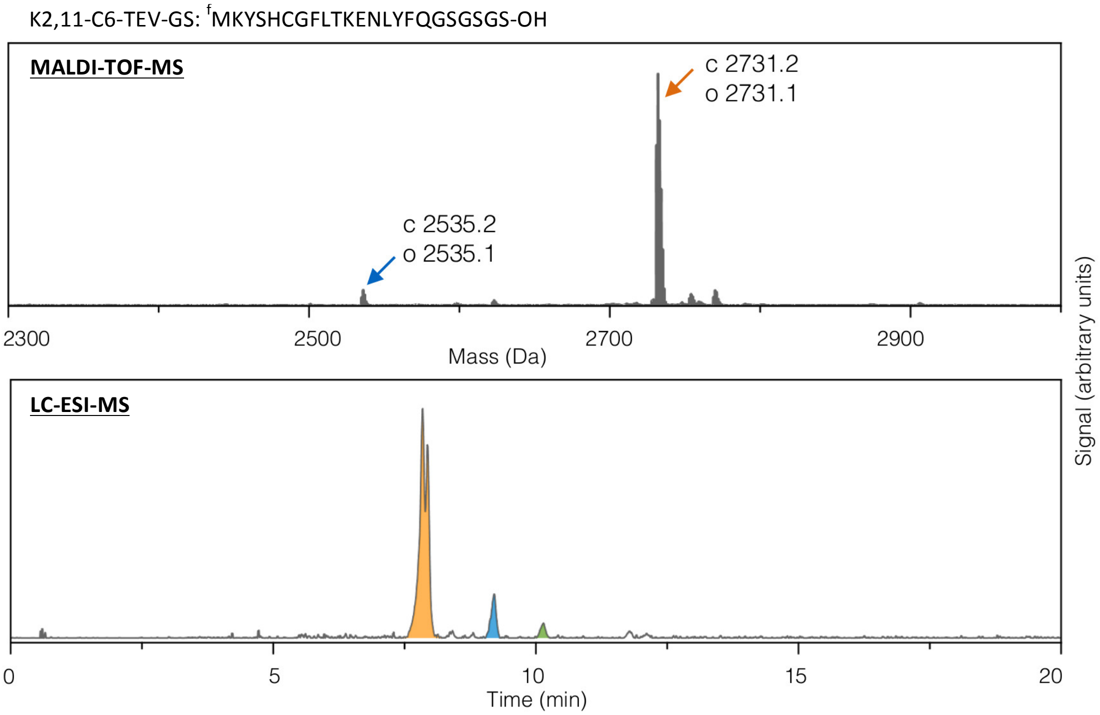

Fig. S9 Corroboration of MALDI-TOF-MS approximation of reaction progress (upper) by ESI-LC-MS data (lower, XIC: 1285.0-1286.5+1268.0-1269.5+1366.0-1367.5 Da, $[M+2H]^{2+}$ for eliminated product, translation product, and conjugate addition product, respectively) for the addition of glucose-1-thiol to the K2,11-C6-TEV-GS peptide. Products are colour coded as in the main text: green for the unmodified translation product, blue for eliminated cysteine, orange for the conjugate addition product.

TIC and supplementary XIC traces for LC-ESI-MS data

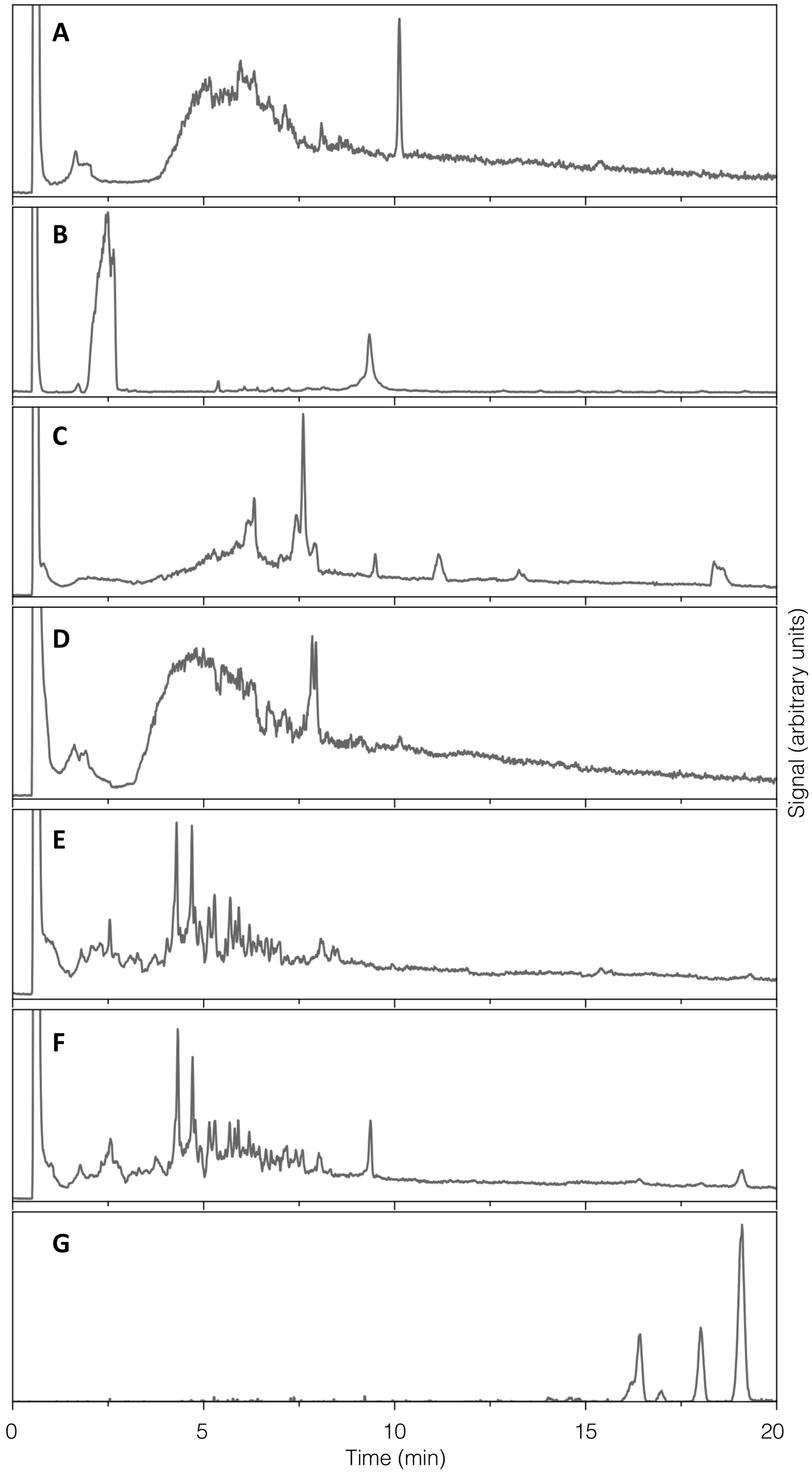

Fig. S10 LC-ESI-MS total ion (500-3000 Da) traces for (A) translation, (B) elimination, (C) conjugate addition of 4-carboxybenzyl thiol, (D) conjugate addition of 1-thio-β-D-glucose, (E) DSG cyclisation then conjugate addition of 1-thio-β-D-glucose, and (F) DSG cyclisation (*cf.* extracted ion traces in Fig. 5). (G) Extracted ion chromatogram ($[M+2H]^{2+}$; 1316.0-1317.5 Da) for DSG cyclisation, showing multiple peaks.

Formation of an *N*-terminal ketone by DBAA

The formation of an *N*-terminal ketone by DBAA was illustrated using the C2,10-TEV-GS test peptide, which was deliberately translated in the absence of an initiating amino acid. This results in translation starting from the second codon (Cys), although typically with lower efficiency. This peptide, with both an internal and an *N*-terminal cysteine, was observed to form an unexpectedly stable internal disulfide bond, meaning that the standard reduction strategy with DTT was insufficient for complete conversion to free cysteines. Longer reduction with a higher concentration of DTT, followed by treatment with DBAA (50 mM) gave rise to two products: an unusually high proportion of cross-linked thiols (*cf.* Fig. 3 and S7), as well as a product 1 Da larger than 2 simple eliminations. This is consistent with the expected rearrangement of an *N*-terminal enamine to an imine, followed by hydrolysis. Further confirmation of ketone formation was derived from reaction with 1-thio-β-d-glucose, which was reactive at only one position, followed by addition of biotin hydrazide under mild acid conditions, which was seen to form an adduct of mass consistent with an *N*-terminal hydrazone, and not with conjugate addition (Fig. S9). The peak arising from cross-linking of cysteines by DBAA was no longer visible after this hydrazide reaction step, but no new peak was visible for its conversion to either starting material or a new side-product. This may be because of poor MALDI ionisation, or precipitation, or formation of a product outside of the MALDI spectral window (for example from reaction with proteins of the translation system). It is also worth noting that biotin hydrazide did not show any signs of reaction with dehydroalanine if added before thiol (data not shown), although the N-terminal hydrazone was observed to form, and the peak from cross-linking of cysteines was again seen to disappear. Hydrazone formation with the *N*-terminal ketone required prolonged incubation and did not reach completion, but changing to a hydroxylamine and adding an aniline catalyst,[4] or using a 2-amino benzamidoxime,[5] should give a more convenient and efficient reaction for selective *N*-terminal modification.

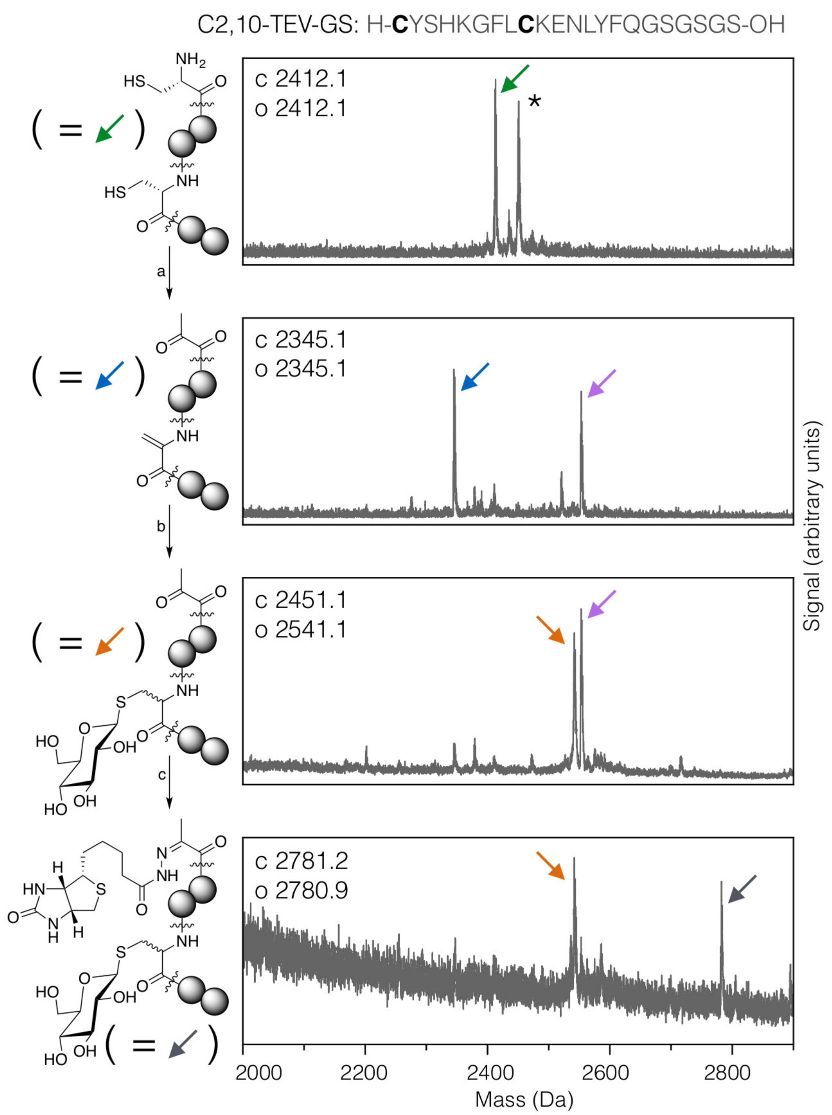

Fig. S11 MALDI-TOF-MS spectra showing the translation, elimination, thiol addition, and hydrazide addition for a peptide containing internal and *N*-terminal cysteines. Shown above the spectrum is the peptide sequence, while to the left is a cartoon semi-structural representation of the key functional groups. Major peaks are colour coded as indicated, with the addition of magenta arrows for cysteines cross-linked with DBAA (as in Fig. S7). Reaction conditions: (a) 50 mM DBAA, pH 8.5 sodium phosphate buffer, 1.5 hours, 42 °C, (b) 42 mM 1-thio-β-D-glucose, pH 8.5 sodium phosphate buffer, 2 hours, 37 °C, (c) 13.75 mM biotin hydrazide, pH 5.2 sodium acetate buffer, 3 hours, 37 °C. * = potassium adduct.

Supplementary references

1 K. Iwasaki, Y. Goto, T. Katoh and H. Suga, *Org. Biomol. Chem.*, 2012, **10**, 5783–5786.

2 Y. Yamagishi, I. Shoji, S. Miyagawa, T. Kawakami, T. Katoh, Y. Goto and H. Suga, *Chem. Biol.*, 2011, **18**, 1562–1570.

3 P. M. Morrison, P. J. Foley, S. L. Warriner and M. E. Webb, *Chem. Commun.*, 2015, **51**, 13470–13473.

4 S. Ng, E. Lin, P. I. Kitov, K. F. Tjhung, O. O. Gerlits, L. Deng, B. Kasper, A. Sood, B. M. Paschal, P. Zhang, C.-C. Ling, J. S. Klassen, C. J. Noren, L. K. Mahal, R. J. Woods, L. Coates and R. Derda, *J. Am. Chem. Soc.*, 2015, **137**, 5248–5251.

5 P. I. Kitov, D. F. Vinals, S. Ng, K. F. Tjhung and R. Derda, *J. Am. Chem. Soc.*, 2014, **136**, 8149–8152.
